# Mode-Dependent Strain-Rate Sensitivity of Filled Polymer Yield Under Shear and Uniaxial Deformation

**DOI:** 10.3390/polym18151823

**Published:** 2026-07-25

**Authors:** Yancai Sun, Feng Shi, Jian Xu, Peiwu Hou, Wenjuan Bai, Dianming Chu, Wenzhong Deng

**Affiliations:** 1 College of Mechanical and Electrical Engineering, Guilin University of Aerospace Technology, Guilin 541004, China; 2024025@guat.edu.cn; 2 College of Mechanical and Electrical Engineering, Qingdao University of Science and Technology, Qingdao 266061, China; 4025037014@mails.qust.edu.cn (P.H.); bwj@qust.edu.cn (W.B.); chudianming@qust.edu.cn (D.C.); 3 University Engineering Research Center of Non-Standard Intelligent Equipment and Process Control Technology, Guangxi, Guilin 541004, China; 4 Academy of Aerospace Solid Propulsion Technology, Xi’an 710025, China; shifengcqu@126.com; 5 Guilin Rubber Industry R&D Institute Co., Ltd., Guilin 541004, China; xujian7@cncec.cn

**Keywords:** polymer nanocomposite, coarse-grained molecular dynamics, non-equilibrium molecular dynamics, strain-rate sensitivity, yield stress, filler de-reinforcement

## Abstract

Filled polymers yield differently under shear and uniaxial deformation, but filler and rate effects remain sparsely characterized. We study a glassy Kremer–Grest polymer with frozen filler beads at N=100, loading ϕ∈[0,0.30], and strain rate $\dot{\varepsilon }\in \left[10^{-4},10^{-2}\right]\;\tau_{\mathrm{LJ}}^{-1}$ by non-equilibrium molecular dynamics. All ten strain-rate-sensitivity confidence intervals exclude zero (uniaxial m∈[0.17,0.30], shear m∈[0.14,0.16]). Uniaxial yield is more rate-sensitive than shear, but the mode gap is loading-dependent: Δm≈0.08–0.16 (p≥0.98) for ϕ≤0.10, attenuating to 0.02–0.04 for ϕ≥0.20 as filler loading increases. Frozen filler softens rather than reinforces (shear yield −78%, plateau modulus −43%); density-matched neat controls attribute most of the shear softening to density dilution (89–97%). At ϕ=0.30, shear develops a low-rate crossover and the yield ratio $\sigma_{y}^{\mathrm{uniax}}/\sigma_{y}^{\mathrm{shear}}$ rises to [1.30,1.40], below the von-Mises reference. Density dilution dominates, but both-mode controls isolate a mode-selective filler contribution at ϕ=0.30 (resolved at the higher rate): 35–42% of the uniaxial versus ∼10% of the shear yield.

## 1. Introduction

Polymer–nanoparticle (NP) composites combine the molecular dexterity of polymer melts with the geometric reinforcement of dispersed filler particles, producing materials whose linear modulus and large-deformation yield response depart from those of the neat polymer in ways that are both quantitatively significant and physically intricate. Industrial applications—from tire rubber, to barrier films, to high-performance elastomers—rely on these departures, yet the molecular-scale mechanisms by which filler loading ϕ modulates the activation pathway of polymer-melt yield remain under-resolved by current coarse-grained molecular dynamics (CG-MD) studies.

Filler-induced reinforcement is canonically measured through the plateau modulus $G_{N}\left(\phi \right)$ as a function of filler volume fraction ϕ, and is well-described in many systems by hydrodynamic-augmented scaling laws (Heinrich–Klüppel, Mooney) or percolation theories [1,2,3]. These hydrodynamic frameworks capture the geometric contribution to reinforcement, while percolation models instead predict a power-law modulus divergence once a filler network forms above a threshold $\phi_{c}$; both presuppose a modulus that grows with loading. In the nonlinear regime, yield stress $\sigma_{y}$ under finite deformation is rate-activated in classical Eyring–Argon frameworks [4,5,6]; the strain-rate sensitivity $m=\partial \ln\sigma_{y}/\partial \ln\dot{\varepsilon }$ is the textbook signature of the activation pathway, growing as deformation shifts from weakly activated, fluid-like segmental flow toward the cooperative, strongly activated rearrangements that characterize glassy yield [5].

These two regimes have, however, been investigated largely in isolation. The linear regime has motivated systematic Green–Kubo extractions of $G_{N}\left(\phi \right)$ in coarse-grained bead-spring nanocomposite simulations [2,7], with the plateau modulus extracted from the long-time decay of the stress autocorrelation function [8,9]. The nonlinear regime has been studied in detail for unfilled bead-spring polymers [10,11,12], for which characteristic strain-hardening regimes and Eyring-type rate dependence have been quantified, and for filled polymer glasses [5]. Recent coarse-grained and atomistic simulations have begun to quantify filler reinforcement and its strain-rate dependence [13], the role of percolated nanoparticle networks [14], and the coupling of cross-link and filler architecture to elastomer mechanics [15]. However, coarse-grained MD studies of filler-induced modulation of yield stress at finite *T*, across both shear and uniaxial deformation modes in the same system, remain sparse. Cross-mode comparison is particularly valuable because it disambiguates two distinct categories of physical mechanism: *continuum-level effects*, (which apply equally to both modes and would yield a constant ratio $\sigma_{y}^{\mathrm{uniax}}/\sigma_{y}^{\mathrm{shear}}=\sqrt{3}$ for an isotropic von-Mises continuum), and *polymer-specific molecular-level effects*, (which couple selectively to chain stretching versus inter-chain sliding and would yield mode-dependent rate dependence at high filler loadings). The mode gap $\Delta m=m_{\mathrm{uniax}}-m_{\mathrm{shear}}$ and the dimensionless yield ratio $r_{y}=\sigma_{y}^{\mathrm{uniax}}/\sigma_{y}^{\mathrm{shear}}$ are accordingly central observables for distinguishing these two categories at the bead-spring level.

In this work, we present a coarse-grained molecular dynamics survey that bridges these regimes for a Kremer–Grest bead-spring glassy polymer with frozen filler beads, building on the bimodal-interphase characterization of our earlier coarse-grained study [16], complemented by our experimental viscoelastic characterization of polymer blends [17]. The dataset combines a 20-system Direction-A plateau-modulus sweep at $T^{*}=1.0$ across N∈{25,50,100} and nominal loading ϕ∈{0.05,…,0.40} where available, together with 150 standard-rate Direction-B NEMD curves (30 cells × 5 seeds) at N=100 across both shear and uniaxial deformation modes, ϕ∈{0.00,0.05,0.10,0.20,0.30}, and three strain rates $\dot{\varepsilon }\in \left\{10^{-4},10^{-3},10^{-2}\right\}\;\tau_{\mathrm{LJ}}^{-1}$. Every cell is sampled with five independent thermal-velocity seeds in both deformation modes, supporting bootstrap confidence intervals on the strain-rate sensitivity m(ϕ) across both modes. Additional Direction-A temperature/KWW records are used only for supporting observations.

Three principal findings emerge. *First*, uniaxial yield is consistently more rate-sensitive than shear yield. With a full three-rate, five-seed sampling at every cell, all ten strain-rate sensitivities are resolved with 95% CIs excluding zero (uniaxial m∈[0.17,0.30], shear m∈[0.14,0.16]); both modes are genuinely rate-strengthening at every loading. The mode gap $\Delta m=m_{\mathrm{uniax}}-m_{\mathrm{shear}}$ is positive at every loading and strongly loading-dependent; it is largest and statistically robust at low loading (p(Δm>0)≥0.98 for ϕ≤0.10) and is progressively attenuated as filler loading increases, falling to within bootstrap scatter at ϕ≥0.20 (Δm≈0.02–0.04, p≈0.64–0.71). We interpret the low-loading asymmetry as tension activating rate-sensitive chain-stretching modes while shear is accommodated partly by lower-barrier inter-chain sliding, and we hypothesize that its high-loading attenuation reflects the increasingly bridged filler network constraining that channels—a mechanism the structural descriptors below are consistent with but do not, on their own, establish. Separately, at the highest loading (ϕ=0.30), the shear yield stress exhibits a rate crossover—rate-independent below $\dot{\varepsilon }\approx 10^{-3}$ and rate-strengthening above—which we report as a distinct high-loading observation. *Second*, the two-dimensional yield-stress ratio $\sigma_{y}^{\mathrm{uniax}}/\sigma_{y}^{\mathrm{shear}}$ falls in [0.80,1.40] across all $\left(\phi ,\dot{\varepsilon }\right)$ cells, in every case below both isotropic-continuum bounds—the von-Mises ($\sqrt{3}\approx 1.73$) and Tresca (2) ratios. For ϕ≤0.20, the ratio stays near unity ($r_{y}\in \left[0.80,1.16\right]$): uniaxial and shear yield are comparable, far below either bound, consistent with inter-chain-sliding-dominated (pressure-sensitive) yielding. It rises to $r_{y}\in \left[1.30,1.40\right]$ only at the highest loading ϕ=0.30 (still below the von-Mises reference $\sqrt{3}$). *Third*, frozen filler produces a counterintuitive softening—here termed *de-reinforcement*—at N=100: the linear plateau modulus and the shear yield stress both decrease monotonically with loading (shear $\sigma_{y}$ by ∼78%, Spearman ρ=−1.00; $G_{N}$ by ∼43%). In the density-matched control set, neat polymer compressed to the filled-box densities recovers most of this softening (89–97%), so matrix-density dilution is the dominant contribution; the filler itself adds only a small high-loading residual. The trend nonetheless remains opposite to the premise of standard reinforcement scaling laws for the present frozen point-LJ inclusion limit. The softening turns mode-selective at the highest loading ϕ=0.30, where the uniaxial yield stress recovers while shear yield keeps falling; density-matched controls run in both deformation modes show this recovery to be a genuine filler contribution—35–42% of the uniaxial yield versus about 10% of the shear yield—not a residual of the changing matrix density.

Together, these findings illustrate that the linear and nonlinear regimes of polymer–NP composites do not collapse onto a single bridging ratio ($\sigma_{y}/\left(G_{N}\gamma_{y}\right)$ varies by ∼30−40% across ϕ). The rate-activated yield observable m(ϕ) operates as an observable orthogonal to the linear plateau modulus, and its mode-selective dependence on filler loading provides a new window for generating and testing hypotheses about how filler loading couples to the activation pathway in glassy polymer–nanoparticle composites. To our knowledge, this is the first coarse-grained study to report, within a single filled bead-spring system, a matched shear-versus-uniaxial, mode-resolved strain-rate sensitivity m(ϕ) with five-seed bootstrap confidence intervals on every *m* and on the mode gap; because this observable is orthogonal to the plateau modulus, it retains a filler-specific signature after matrix-density dilution is controlled for—a signature that density-matched controls in both deformation modes localize here to the ϕ=0.30 deviatoric (uniaxial) response, where the filler contributes 35–42% of the yield.

## 2. Materials and Methods

### 2.1. System and Force Field

Coarse-grained molecular dynamics simulations were performed in LAMMPS (version 22 July 2025, Update 1) [18,19], inheriting the polymer–nanocomposite geometry of our earlier study [16]. The system comprises a dense bead-spring polymer of $N_{\mathrm{chain}}=80$ linear chains with N=100 Kremer–Grest bead-spring beads each [20,21] (type 1, $m_{1}=1$) and a number of frozen type-2 filler beads (type 2, $m_{2}=10$) chosen to set the nominal filler loading ϕ∈{0.00,0.05,0.10,0.20,0.30}. Throughout, ϕ denotes the nominal loading defined by the geometric convention of [16] (notional filler diameter $\sigma_{f}=3.0\;\sigma$), and is proportional to the filler number density rather than to a hard-sphere volume fraction in the present point-LJ representation ($\sigma_{2-2}=\sigma_{1-2}=1$); the linear scaling of filler count with ϕ is verified in Section 4.6. We retain the symbol ϕ for continuity with [16] but interpret it as a loading index, not a strict volume fraction.

All non-bonded interactions use a Lennard–Jones potential:

(1) $$U_{\mathrm{LJ}}\left(r\right)=4\varepsilon \left[{\left(\frac{\sigma }{r}\right)}^{12}-{\left(\frac{\sigma }{r}\right)}^{6}\right],\;r<r_{\mathrm{cut}}=2.5\;\sigma ,$$

with ε=σ=1 (LJ units) for all type pairs (1–1, 2–2, 1–2; no mixing-rule asymmetry) and an energy shift to zero at $r_{\mathrm{cut}}$ (pair_modify shift yes). Chain connectivity is enforced by harmonic bonds $U_{\mathrm{bond}}\left(r\right)=\frac{1}{2}K{\left(r-r_{0}\right)}^{2}$ with $K=500\;\varepsilon /\sigma^{2}$ and $r_{0}=0.97\;\sigma$, which suppresses bond crossings without invoking FENE non-linearity. Filler particles are immobile during all dynamics phases (fix freeze filler setforce 0 0 0); this convention, established in [16], cleanly partitions the mechanical response between polymer dynamics and filler-induced kinematic constraint. The simulation cell is periodic in all three Cartesian directions; box edge lengths range from 16.08σ (rebuilt neat) to 18.00σ at ϕ=0.30, corresponding to a bead number density $\rho \approx 1.93\;\sigma^{-3}$ for the neat system, inherited from the NPT density-correction protocol of [16]. This density is well above the canonical Kremer–Grest value ($\rho \approx 0.85\;\sigma^{-3}$), and at $T^{*}=1.0$ the monomer dynamics are kinetically arrested: an equilibrium diagnostic run on the neat configuration gives a mean-square displacement that plateaus near $0.06\;\sigma^{2}$ over $540\;\tau_{\mathrm{LJ}}$ (root-displacement ≈0.24σ, effective self-diffusion $D\approx 4\times 10^{-6}\;\sigma^{2}/\tau_{\mathrm{LJ}}$); an analogous run at ϕ=0.30 (the least dense cell, $\rho \approx 1.42\;\sigma^{-3}$) yields a comparable plateau ($\mathrm{MSD}\approx 0.08\;\sigma^{2}$), confirming caged dynamics across the full filler range. The systems are therefore best described as *dense, kinetically arrested (glassy) bead-spring solids* rather than entangled liquid melts; we adopt this characterization throughout and interpret the yield response within the Eyring–Argon activated-deformation framework for polymer glasses [4,5], which is the appropriate regime for caged segmental dynamics. All temperatures reported here are $T^{*}=k_{B}T/\varepsilon =1.0$. Under the conventional Kremer–Grest mapping (5–10 backbone monomers per bead, σ≈0.5nm), each chain represents 500–1000 backbone atoms; the approximate nature of this mapping is discussed in Section 4.6.

### 2.2. Equilibration Protocol

Equilibrated initial configurations are inherited from the five-phase protocol in [16]: (i) soft push-off, ramping a soft potential from 0 to 300 over 60,000 steps at Δt=0.001τ; (ii) switch to the full LJ potential with brief NVE/NVT equilibration; (iii) NPT density correction (500,000 steps); (iv) *N*-dependent NVT equilibration ($1.0\times 10^{7}$ steps for N=100); and (v) NVT production. Before each NEMD deformation run, an additional brief NVT re-equilibration is performed at $T^{*}=1.0$ (fix nvt polymer temp 1.0 1.0 1.0, Δt=0.005τ, $4\times 10^{4}$ steps $\equiv 200\;\tau_{\mathrm{LJ}}$); this serves to thermalize polymer velocities after configuration loading and to allow brief stress relaxation following any tilt-box change.

### 2.3. Linear Viscoelastic Measurement (Green–Kubo)

Linear-regime stress relaxation moduli G(t) are obtained from the Green–Kubo relation:

(2) $$G\left(t\right)=\frac{V}{k_{B}T}\frac{1}{3}\sum_{\alpha \neq \beta }\langle \sigma_{\alpha \beta }\left(0\right)\;\sigma_{\alpha \beta }\left(t\right)\rangle ,$$

averaging over the three off-diagonal stress components αβ∈{xy,xz,yz} to maximize statistical efficiency. The stress autocorrelation is accumulated on-the-fly via LAMMPS fix ave/correlate and sampled out to $t\approx 2\times 10^{4}\;\tau_{\mathrm{LJ}}$ over the production trajectory. The plateau modulus $G_{N}$, the linear-viscoelastic signature of the entanglement/packing network [22], is extracted as the time-average of the normalized G(t) over the long-time window $t\in \left[5000,\;19,990\right]\;\tau_{\mathrm{LJ}}$ (1500 sampled points), which lies past the fast short-time (β-like) decay (envelope sampled over $t\in \left[10,100\right]\;\tau_{\mathrm{LJ}}$) yet within the arrested plateau at this density.

The linear modulus is quantified by the dimensionless ratio $R_{\mathrm{reinforce}}\left(N,\phi \right)=G_{N}\left(\phi \right)/G_{N}\left(\phi_{\min}\right)$ where $\phi_{\min}=0.05$ is the lowest filler loading in our sweep. We compare the resulting $G_{N}\left(\phi \right)$ against the standard reinforcement laws—the Heinrich–Klüppel hydrodynamic prediction $R=1+2.5\phi_{\mathrm{eff}}+14.1\phi_{\mathrm{eff}}^{2}$ [1], the power-law $R=1+A\phi^{x}$, percolation $R={\left(\phi /\phi_{c}\right)}^{t}$, and the Mooney correction $R=\exp[A\phi /\left(1-\phi /\phi_{m}\right)]$—each of which presupposes a modulus that increases with loading and is therefore inapplicable to the observed de-reinforcement (see Section 3).

### 2.4. Non-Equilibrium MD: SLLOD Simple Shear

Simple shear deformation is imposed via the SLLOD algorithm [11,23,24], which couples a streaming velocity profile $v_{x}=\dot{\gamma }\;y$ to a thermostat acting on peculiar velocities only. After equilibration, the orthorhombic box is converted to triclinic (change_box all triclinic); the tilt factor xy is then driven at constant engineering strain rate $\dot{\gamma }$ through fix deform 1 xy erate $\dot{\gamma }$ remap v. The polymer group is thermostatted with fix nvt/sllod temp 1.0 1.0 1.0 (damping time $\tau_{T}=1.0\;\tau_{\mathrm{LJ}}$, profile-aware velocity removal); filler particles remain immobile. Three strain rates are spanned, $\dot{\gamma }\in \left\{10^{-4},10^{-3},10^{-2}\right\}\;\tau_{\mathrm{LJ}}^{-1}$, each integrated to a nominal accumulated strain $\gamma_{\max}=1.0$ ($2\times 10^{6}$, $2\times 10^{5}$, and $2\times 10^{4}$ steps respectively, Δt=0.005τ). The engineering shear stress $\sigma_{xy}\left(\gamma \right)=p_{xy}$ is sampled every 1000 steps, yielding ∼200 output points per simulation at $\dot{\gamma }=10^{-3}$ and proportionally more at slower rates. Five independent thermal-velocity seeds (s∈ {17239, 28471, 39827, 51803, 64219}), which differ only in the initial Maxwell–Boltzmann velocity draw from a single equilibrated configuration per ϕ (Section 2.10), provide replicate sampling for every standard $\left(\phi ,\dot{\gamma }\right)$ cell; they therefore sample thermal (dynamical) variability at fixed filler geometry rather than independent quenched filler realizations. Additional intermediate shear rates ($3\times 10^{-4}$ and $3\times 10^{-3}$ where used for the crossover diagnostic) are sampled with two seeds and are excluded from the standard three-rate m(ϕ) fits.

### 2.5. Non-Equilibrium MD: Uniaxial Extension

Uniaxial extension is imposed by fix deform 1 x erate $\dot{\varepsilon }$ remap x, driving the *x* box edge at constant engineering strain rate while *y* and *z* edges remain fixed; the polymer group is thermostatted with fix nvt (the SLLOD profile-removal is not required for pure extension since the streaming profile is uniform). The deviatoric stress is computed as $\sigma_{\mathrm{dev}}=\sigma_{xx}-\frac{1}{2}\left(\sigma_{yy}+\sigma_{zz}\right)$, the axial deviatoric (von-Mises-type) stress measure. Holding the *y* and *z* edges fixed makes this confined uniaxial *strain* ($\varepsilon_{yy}=\varepsilon_{zz}=0$) rather than free uniaxial *stress* (see Section 4.6). The same rate matrix $\dot{\varepsilon }\in \left\{10^{-4},10^{-3},10^{-2}\right\}$ is spanned, with seed coverage matched to the shear runs.

### 2.6. Yield-Stress Extraction, Von-Mises/Tresca Framework, and Rate-Sensitivity Fitting

For the yield observables used here ($\sigma_{y}^{\mathrm{shear}}=\max\left|\sigma_{xy}\right|$ and $\sigma_{y}^{\mathrm{uniax}}=\max\left|\sigma_{\mathrm{dev}}\right|$, with $\sigma_{\mathrm{dev}}$ the axial deviatoric stress), an isotropic elastic–plastic continuum predicts a uniaxial-to-shear yield ratio of $\sigma_{y}^{\mathrm{uniax}}/\sigma_{y}^{\mathrm{shear}}=\sqrt{3}\approx 1.73$ under the von-Mises (maximum-distortional-energy) criterion and $\sigma_{y}^{\mathrm{uniax}}/\sigma_{y}^{\mathrm{shear}}=2$ under the Tresca (maximum-shear-stress) criterion; von-Mises is thus the lower of the two continuum bounds, and both exceed unity. A measured ratio $r_{y}=\sigma_{y}^{\mathrm{uniax}}/\sigma_{y}^{\mathrm{shear}}\approx 1$ therefore indicates near-equal uniaxial and shear yield, well below either isotropic-continuum bound—as expected when yielding is governed by inter-chain sliding (and is pressure-sensitive) rather than by $J_{2}$ deviatoric flow. For each curve, we report two yield-stress observables. The *peak yield* $\sigma_{y}^{\mathrm{peak}}$ is the maximum of |σ| in the monotonic forward-loading window $\gamma_{\mathrm{eng}}\in \left[0,0.5\right]$ (for shear) or $\varepsilon_{\mathrm{eng}}\in \left[0,0.5\right]$ (for tension); this window deliberately excludes the bidirectional tilt-box pre-shear region γ<0 where occasional sharp transient stress events were observed at ϕ=0.20 (see Supplementary Materials Figure S1). The *small-yield reference* $\sigma_{y}^{0.1}$ is the mean |σ| over γ∈[0.08,0.12] (or ε∈[0.08,0.12]); this fixed-strain definition is more robust to peak-position noise and is the basis of the rate-sensitivity analysis below. We treat the resulting *m* as an operational small-strain rate sensitivity, not a fundamental activation volume, and read its three-rate estimate as a trend rather than a calibrated activation parameter.

The strain-rate sensitivity

(3) $$m=\frac{\partial \ln\sigma_{y}^{0.1}}{\partial \ln\dot{\varepsilon }}$$

is extracted per (ϕ, mode) by the linear regression of $\ln\sigma_{y}^{0.1}$ on $\ln\dot{\varepsilon }$ across the three strain rates, after seed-averaging $\sigma_{y}^{0.1}$ at each $\dot{\varepsilon }$. To propagate the seed-level uncertainty into the *m* estimate, we perform a parametric Monte Carlo bootstrap [25] with $N_{\mathrm{MC}}=1000$ iterations: at each iteration, $\sigma_{y}^{0.1}$ is resampled at each $\dot{\varepsilon }$ from a Gaussian $N(\langle \sigma_{y}^{0.1}\rangle ,s_{\sigma_{y}^{0.1}})$, with $s_{\sigma_{y}^{0.1}}$ the five-seed standard deviation at each strain rate. Because the five seeds share a common equilibrated configuration at each ϕ, these intervals propagate thermal (seed-level) variability at fixed filler geometry and do not include configurational (filler-packing) uncertainty; the consequences for interpretation are discussed in the Limitations. The 95% confidence interval on *m* is reported as the 2.5–97.5% quantile of the bootstrap distribution. The mode gap is assessed by a *paired* bootstrap ($N_{\mathrm{MC}}=5000$, fixed seed): at each iteration, the shear and uniaxial slopes are refit from independently resampled $\sigma_{y}^{0.1}$ and differenced to form $\Delta m=m_{\mathrm{uniax}}-m_{\mathrm{shear}}$, from which the 95% CI and the exceedance probability p(Δm>0) are computed; this tests the mode difference directly rather than inferring it from the overlap of the two single-mode intervals. Whether the gap changes between low and high loading is tested analogously by bootstrapping Δm(ϕ=0.30)−〈Δm(ϕ≤0.20)〉.

### 2.7. Linear–Nonlinear Bridging Analysis

To test whether linear modulus solely controls the nonlinear yield response, we compute the dimensionless reduced yield strain $\sigma_{y}^{\mathrm{peak}}/\left(G_{N}\;\cdot \;\gamma_{y}\right)$ for combinations $\left(\phi ,\dot{\varepsilon }\right)$ where both Direction-A linear $G_{N}\left(\phi \right)$ and Direction-B NEMD yield data are available. Three normalizations are tested: $\gamma_{y}=$ engineering yield strain at peak; $\gamma_{y}=0.1$ (fixed small-strain reference); and the simple ratio $\sigma_{y}/G_{N}$. A coefficient of variation (CV) of the reduced strain across ϕ at fixed (mode, $\dot{\varepsilon }$) is used as the diagnostic of ϕ-independence; CV <0.1 would indicate that linear modulus and a fixed yield-strain ratio fully determine the nonlinear response.

### 2.8. Filler Connectivity and Interphase Metrics

To characterize the filler contact network that the rate- and mode-response analysis invokes, we compute two static structural descriptors on the equilibrated configurations. For each polymer chain, we count the distinct filler beads within a contact cutoff $r_{c}=1.5\;\sigma$; the *chain-bridging fraction* is the fraction of chains contacting two or more distinct fillers, and the *interphase fraction* is the fraction of polymer beads lying within $r_{c}$ of any filler. These are connectivity descriptors of the frozen filler contact network, not a finite-size-scaled percolation transition; we accordingly report a bridging-*saturation* loading (the label at which the chain-bridging fraction reaches 100%) rather than a percolation threshold with critical exponents, and we use these metrics only to locate, not to explain, the high-loading regime.

### 2.9. KWW Fitting Protocol (Direction A)

For the normalized Green–Kubo curves $\hat{G}\left(t\right)=G\left(t\right)/G\left(0\right)$, a stretched-exponential (Kohlrausch–Williams–Watts) plus plateau form is fit [26]:

(4) $$\hat{G}\left(t\right)=A_{\mathrm{plateau}}+\left(1-A_{\mathrm{plateau}}\right)\exp\;\left[-{\left(t/\tau_{\mathrm{KWW}}\right)}^{\beta_{\mathrm{KWW}}}\right],$$

via SciPy curve_fit with bounded least squares ($\tau_{\mathrm{KWW}}\in \left[10^{-1},10^{4}\right]\;\tau_{\mathrm{LJ}}$, $\beta_{\mathrm{KWW}}\in \left[0.05,1.0\right]$, $A_{\mathrm{plateau}}\in \left[0,1\right]$). The fit window covers the trusted decay range identified by the running-noise detector of [16]. We adopt $R^{2}>0.7$ as the nominal fit-quality threshold; 5/24 of the earlier systems satisfy this criterion. The KWW parameters are reported as supporting data in the Supplementary Materials rather than as a primary observable, given their sparse fit success.

### 2.10. Reproducibility

All LAMMPS input scripts, equilibrated start configurations (start_phi*.data), and Python 3.9.6 analysis scripts (NumPy, SciPy, Matplotlib) are prepared for the supplementary repository. The post-equilibration analysis scripts (aggregate_nemd.py, fit_yield_eyring.py, fit_R_reinforce_scaling.py, bridge_linear_nonlinear.py, kww_fit.py) are fully deterministic; the only stochastic component is the Monte Carlo bootstrap, which uses fixed seed 20260528. Total NEMD wall time was approximately 50 h for the full 150-curve five-seed matrix (14 CPU cores, 8 MPI ranks per simulation). To document configuration provenance, the five starting configurations (ϕ=0,0.05,0.10,0.20,0.30) are verified pairwise-distinct by MD5 checksum and by structural fingerprint (filler count 0,35,74,166,285; number density $\rho =1.93,1.86,1.69,1.55,1.42\;\sigma^{-3}$; single-chain radius of gyration $R_{g}=9.3$–12.4σ); the full checksum/fingerprint table is provided in the Supplementary Materials. There is a single equilibrated configuration per ϕ, and the five NEMD seeds re-draw only the initial velocities from it; the configurational (filler-packing) ensemble is thus sampled at one realization per loading, a scope we address in Section 4.6. The ϕ=0 neat baseline was rebuilt for this study (Limitation iii) and is distinct from all filled configurations. All manuscript figures, numerical overlays, labels, and scientific interpretations derive from the author-verified scripts and data files described above.

## 3. Results

### 3.1. Dataset Overview

**Figure 1:**
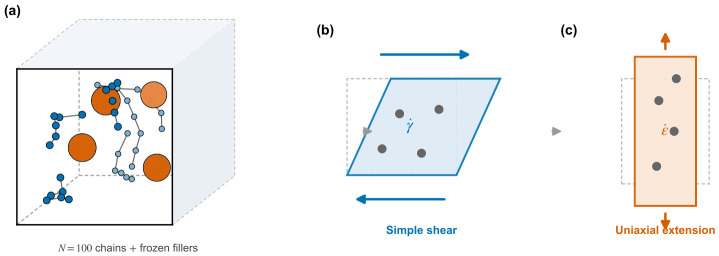
System and deformation-protocol diagram for the coarse-grained filled bead-spring polymer. (**a**) Kremer–Grest bead-spring chains (N=100, type-1 polymer beads connected by harmonic bonds $K=500\;\varepsilon /\sigma^{2}$, $r_{0}=0.97\;\sigma$) and frozen type-2 filler beads in a periodic cubic cell at nominal loading ϕ∈{0.00,0.05,0.10,0.20,0.30}. (**b**) Direction-B SLLOD simple shear via tilt-box xy at constant $\dot{\gamma }$. (**c**) Uniaxial extension via *x*-box deformation at constant $\dot{\varepsilon }$. Periodic boundary conditions apply in all three directions. In (**a**), type-1 polymer beads are blue and frozen type-2 filler beads orange; in (**b**,**c**) the fillers are grey circles, with the simple-shear cell outlined in blue and the extension cell in orange. The colors are schematic and carry no quantitative meaning. Diagram only (illustrative configuration); no data source.

**Figure 2:**
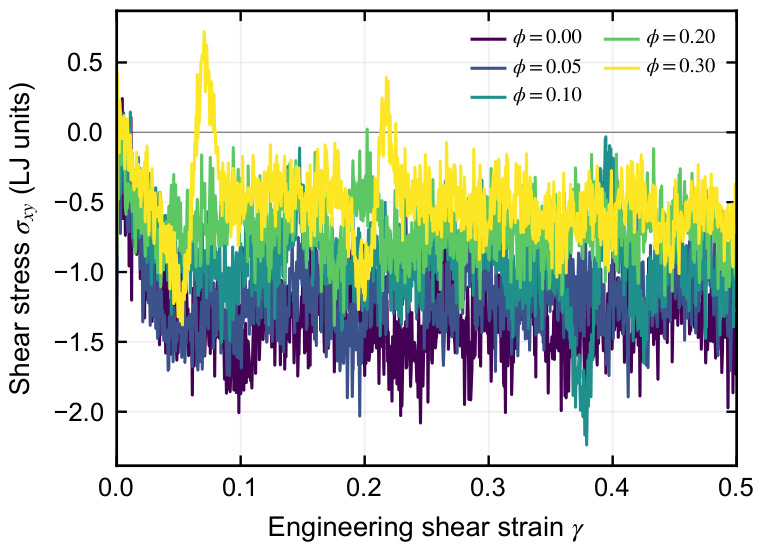
Shear stress–strain response $\sigma_{xy}\left(\gamma \right)$ at ϕ∈{0.00,0.05,0.10,0.20,0.30} for the slowest strain rate $\dot{\gamma }=10^{-4}\;\tau_{\mathrm{LJ}}^{-1}$, N=100, $T^{*}=1.0$. Curves show the forward-loading window γ∈[0,0.5]. The peak yield $\sigma_{y}^{\mathrm{peak}}$ extracted from this window and the small-yield value $\sigma_{y}^{0.1}$ at γ=0.10 form the inputs to the Eyring analysis. The horizontal grey line marks zero stress ($\sigma_{xy}=0$).

**Figure 3:**
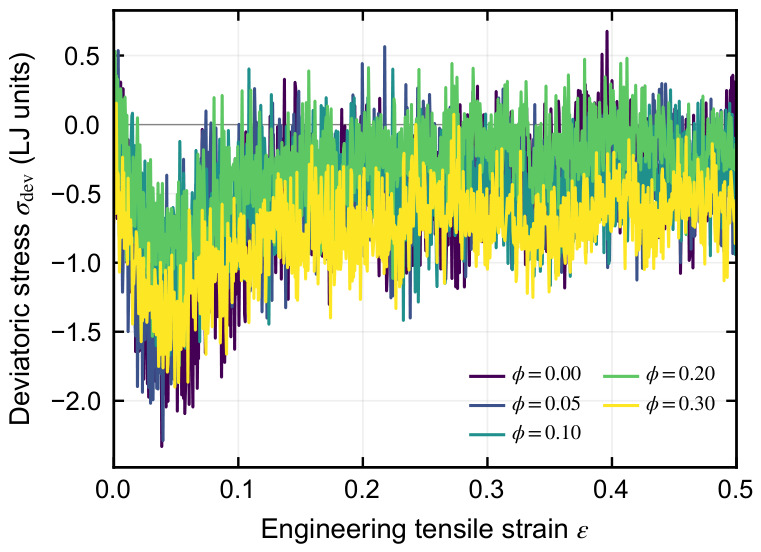
Uniaxial deviatoric stress–strain response $\sigma_{\mathrm{dev}}\left(\varepsilon \right)$ at ϕ∈{0.00,0.05,0.10,0.20,0.30} for the slowest extension rate $\dot{\varepsilon }=10^{-4}\;\tau_{\mathrm{LJ}}^{-1}$, N=100, $T^{*}=1.0$. The deviatoric proxy $\sigma_{\mathrm{dev}}=\sigma_{xx}-\frac{1}{2}\left(\sigma_{yy}+\sigma_{zz}\right)$ isolates the Tresca-/von-Mises-type yield observable. The horizontal grey line marks zero stress ($\sigma_{\mathrm{dev}}=0$).

The system geometry is illustrated in Figure 1: a Kremer–Grest bead-spring polymer with frozen type-2 filler beads, subjected to either simple-shear (SLLOD) or uniaxial deformation NEMD protocols. The full dataset comprises 20 Direction-A plateau-modulus systems at $T^{*}=1.0$ (N∈{25,50,100}, ϕ∈{0.05,…,0.40} where available), two additional Direction-A temperature-sweep points used as supporting observations, and 150 Direction-B standard-rate large-deformation NEMD curves (75 shear + 75 uniaxial; ϕ∈{0.00,0.05,0.10,0.20,0.30} at N=100, $T^{*}=1.0$, $\dot{\varepsilon }\in \left\{10^{-4},10^{-3},10^{-2}\right\}$, five seeds per cell in both modes). The neat (ϕ=0) baseline comprises 30 curves generated from a purpose-built filler-free configuration (see Section 2); the 120 filled-system shear and uniaxial curves (ϕ=0.05–0.30) are from the supplementary simulation campaign. A further six intermediate-rate shear simulations (two seeds each at $\dot{\varepsilon }=3\times 10^{-4}$ and $3\times 10^{-3}$ for ϕ=0.30, and at $3\times 10^{-3}$ for the ϕ=0.20 control) resolve the high-loading rate crossover analyzed in Section 4. Coverage is summarized in Table 1. Representative shear and uniaxial stress–strain responses at the slowest rate are shown in Figure 2 and Figure 3. Both the shear and uniaxial matrices are closed across all five ϕ at every rate with five seeds per cell; this five-seed coverage anchors the rate-sensitivity analysis below.

### 3.2. Mode-Dependent Strain-Rate Sensitivity m(ϕ) is the Primary Observable

**Figure 4:**
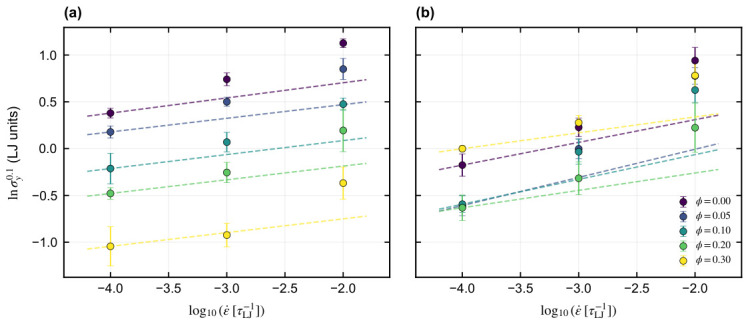
Eyring fits $\ln\sigma_{y}^{0.1}$ versus $\ln\dot{\varepsilon }$ at each ϕ for (**a**) shear and (**b**) uniaxial deformation. Error bars on data points are the five-seed standard deviation; shaded bands represent the bootstrap 95% CI on the linear fit. The slope of each line is the strain-rate sensitivity m(ϕ) reported in Table 2.

**Figure 5:**
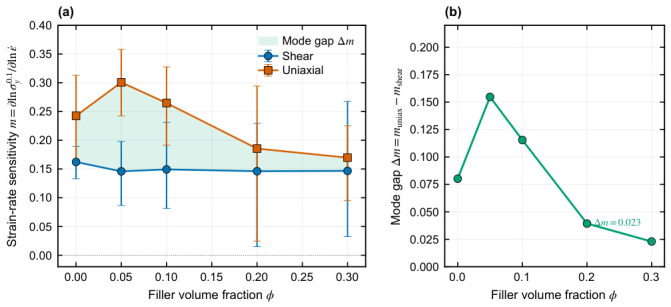
**Main finding**. (**a**) Strain-rate sensitivity m(ϕ) for shear (blue) and uniaxial (orange) deformation, from a full three-rate, five-seed sampling at every cell. Error bars are the bootstrap 95% confidence interval (Table 2). Both branches are rate-sensitive at all filler loadings—uniaxial m∈[0.17,0.30], shear m∈[0.14,0.16]—with all ten CIs strictly above zero; neither branch collapses to rate-independence. (**b**) The mode gap $\Delta m=m_{\mathrm{uniax}}-m_{\mathrm{shear}}$ is positive at every ϕ but strongly loading-dependent: largest at low loading and progressively attenuated as filler is added, falling to within bootstrap scatter at ϕ≥0.20 (p(Δm>0)≈0.64–0.71), i.e., a mode asymmetry that diminishes with filler loading rather than being independent of it.

The strain-rate sensitivity $m=\partial \ln\sigma_{y}^{0.1}/\partial \ln\dot{\varepsilon }$ (Equation (3)), extracted independently for shear and uniaxial deformation across ϕ∈{0.00,0.05,0.10,0.20,0.30} from Eyring-type fits of the yield stress against $\ln\dot{\varepsilon }$ (Figure 4), is the central observable of this work. Table 2 and Figure 5 report point estimates together with 95% bootstrap confidence intervals. Two qualitative features dominate.

*First*, uniaxial *m* exceeds shear *m* at every filler loading by a factor of ∼1.1−2.1 (Table 2), most strongly at low loading. Both branches are fully resolved with five independent thermal-velocity seeds per cell: all five uniaxial cells exhibit m∈[0.17,0.30] and all five shear cells exhibit m∈[0.14,0.16], with every one of the ten 95% confidence intervals strictly excluding zero (e.g., $m_{\mathrm{shear}}\left(\phi =0.30\right)=0.147$, 95% CI [0.033,0.268]; $m_{\mathrm{uniax}}\left(\phi =0.30\right)=0.170$, 95% CI [0.095,0.225]). Both shear and uniaxial yield are therefore genuinely rate-strengthening at all filler loadings; neither branch collapses to rate-independence.

*Second*, the mode gap $\Delta m=m_{\mathrm{uniax}}-m_{\mathrm{shear}}$ is positive at every filler loading, but its magnitude is strongly loading-dependent. A paired parametric bootstrap on the difference (5000 iterations, resampling each rate’s $\sigma_{y}$ and refitting both slopes; see Section 2) gives Δm=0.081,0.155,0.116,0.040, and 0.023 for ϕ=0,0.05,0.10,0.20, and 0.30. The gap is largest and statistically robust at low loading (p(Δm>0)≥0.98 for ϕ≤0.10) and is progressively attenuated as filler is added: at ϕ≥0.20, it drops to Δm≈0.02–0.04 and is no longer resolved against bootstrap scatter (p=0.71 and 0.64 at ϕ=0.20 and 0.30; an onset test returns only p=0.11 that the gap exceeds its low-ϕ baseline at ϕ=0.30). The defensible finding is therefore that uniaxial yielding is more rate-sensitive than shear yielding—robustly so at low filler loading, with the asymmetry falling to statistical insignificance as filler loading increases, over the range where the filler contact network becomes fully bridged. Because Δm at each ϕ is a paired within-configuration comparison—the shear and uniaxial runs share the identical frozen-filler realization—filler-packing variability largely cancels in the mode gap, so the mode asymmetry is robust to the single-realization sampling even where the absolute *m* values carry configurational uncertainty (Section 4.6). We discuss the molecular origin of this loading-dependent asymmetry, and a distinct rate-crossover that emerges only at the highest loading, below.

The Eyring fit on the peak-yield observable $\sigma_{y}^{\mathrm{peak}}$ gives broadly consistent rate dependence but with weaker statistical resolution than $\sigma_{y}^{0.1}$ because peak position varies non-monotonically with $\dot{\varepsilon }$ for several cells; we therefore retain $\sigma_{y}^{0.1}$ as the primary fitting observable and report $\sigma_{y}^{\mathrm{peak}}$ Eyring values in the Supplementary Materials.

### 3.3. Two-Dimensional Yield Surface: $\sigma_{y}^{\mathrm{uniax}}$ Versus $\sigma_{y}^{\mathrm{shear}}$

**Figure 6:**
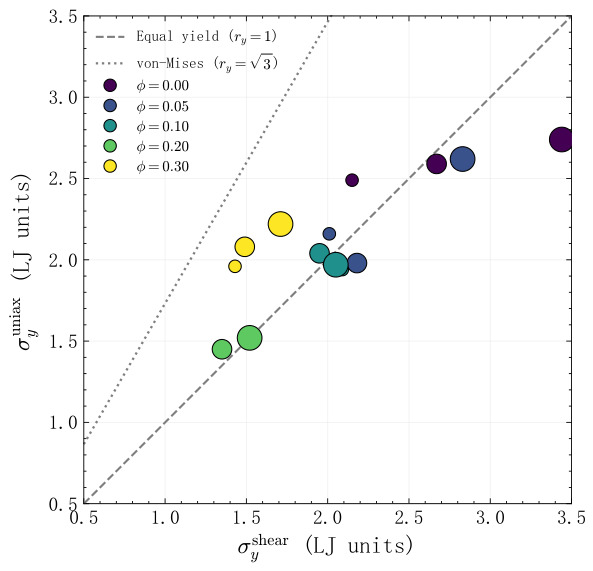
Two-dimensional yield surface: $\sigma_{y}^{\mathrm{uniax}}$ versus $\sigma_{y}^{\mathrm{shear}}$ for all fifteen $\left(\phi ,\dot{\varepsilon }\right)$ cells (five filler loadings × three strain rates), each seed-averaged over five independent seeds (Table 3); color encodes ϕ and symbol size scales with $\log_{10}\dot{\varepsilon }$. Dashed line: equal-yield reference $\sigma_{y}^{\mathrm{uniax}}=\sigma_{y}^{\mathrm{shear}}$ ($r_{y}=1$). Dotted line: von-Mises prediction $\sigma_{y}^{\mathrm{uniax}}=\sqrt{3}\sigma_{y}^{\mathrm{shear}}$ ($r_{y}=\sqrt{3}\approx 1.73$); the Tresca (maximum-shear-stress) ratio for these observables is $r_{y}=2$. All cells lie below the von-Mises reference line ($r_{y}\in \left[0.80,1.40\right]$); the ϕ=0.30 cells have the highest $r_{y}$, still below the reference.

Across all fifteen seed-averaged $\left(\phi ,\dot{\varepsilon }\right)$ cells (Table 3 and Figure 6), the ratio $\sigma_{y}^{\mathrm{uniax}}/\sigma_{y}^{\mathrm{shear}}$ falls between 0.80 and 1.40. Every value lies below both isotropic-continuum bounds—the von-Mises ratio $\sqrt{3}\approx 1.73$ and the Tresca ratio 2. The ratio is, however, loading-dependent: for ϕ≤0.20 it stays near unity ($r_{y}\in \left[0.80,1.16\right]$), i.e., uniaxial and shear yield are comparable and well below either continuum bound, whereas, at the highest loading ϕ=0.30, it rises to $r_{y}\in \left[1.30,1.40\right]$ (still below the von-Mises reference value $\sqrt{3}$)—the same loading at which the shear-yield rate crossover appears, the mode gap closes, and the uniaxial yield stress recovers. We interpret the low-ϕ behavior ($r_{y}\approx 1$, far below the $J_{2}$ and maximum-shear-stress continuum values) as evidence that bulk yield in these glassy polymer–NP composites is dominated by inter-chain sliding and is pressure-sensitive rather than $J_{2}$-deviatoric, with the high-loading bridged filler network at ϕ=0.30 contributing additional deviatoric stress (isolated by the density-matched uniaxial control; see Section 3.4). Independent calibration of the yield criterion in experimental polymer melts at comparable strain rates is sparse, and we frame this 2D mapping as a baseline against which future experimental or atomistic simulation data can be compared.

### 3.4. Frozen Filler De-Reinforces the Matrix: Linear Modulus and Shear Yield Both Decrease

**Figure 7:**
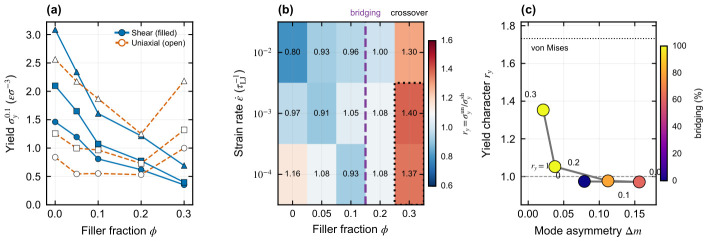
De-reinforcement and the bridging synthesis at N=100. (**a**) Direction-B NEMD yield stress $\sigma_{y}^{0.1}\left(\phi \right)$: shear (filled blue markers) decreases strictly monotonically with loading at every rate (Spearman ρ=−1.00), while the uniaxial yield (open orange markers) de-reinforces up to ϕ=0.20 and recovers at ϕ=0.30; marker shape encodes strain rate (circle $10^{-4}$, square $10^{-3}$, triangle $10^{-2}\;\tau_{\mathrm{LJ}}^{-1}$). (**b**) Yield-ratio regime map $r_{y}=\sigma_{y}^{\mathrm{uniax}}/\sigma_{y}^{\mathrm{shear}}$ over the (filler loading, strain rate) plane (discrete 5×3 samples): near-parity ($r_{y}≲1$, blue) for ϕ≤0.20 and elevated ($r_{y}$ rising but staying below the reference $\sqrt{3}$, red) at ϕ=0.30; the dashed line marks the bridging-saturation loading (chain-bridging fraction reaching 100% at ϕ=0.20) and the dotted box the shear rate-crossover. (**c**) The material’s trajectory through the $\left(\Delta m,r_{y}\right)$ mechanical state space as ϕ increases 0→0.30, colored by the chain-bridging fraction: the path runs from a mode-asymmetric, near-parity ($r_{y}\approx 1$) neat melt to a mode-symmetric, higher-$r_{y}$, fully-bridged composite. The linear modulus $G_{N}$ softens comparably (∼43%; Supplementary Materials Table S3).

At the chain length used throughout the nonlinear survey (N=100), frozen filler produces a counterintuitive softening rather than reinforcement; we refer to this decrease with added filler as *de-reinforcement*. The Direction-A Green–Kubo plateau modulus $G_{N}\left(\phi \right)$ decreases with loading—monotonically once a single ϕ=0.25 single-seed outlier is set aside (Spearman ρ=−0.90, p=0.04), falling ∼43% from ϕ=0.05 to ϕ=0.30 (Supplementary Materials Table S3). The Direction-B NEMD shear yield stress $\sigma_{y}^{0.1}\left(\phi \right)$ decreases more cleanly, strictly monotonically with ϕ at every strain rate (Spearman ρ=−1.00, p<0.001 at each of $\dot{\varepsilon }=10^{-4},10^{-3},10^{-2}$), by 74–81% over the filler range (∼78% averaged across the three rates; Figure 7).

Because the filled configurations also become less dense ($\rho^{*}=1.926$ at ϕ=0 to 1.421 at ϕ=0.30, a 26% reduction), we tested how much of the softening is a density (dilution) effect rather than a genuine filler-network effect. We ran neat-polymer shear NEMD controls at the polymer densities of the ϕ=0.20 and ϕ=0.30 filled boxes (Table 4; per-seed values in Supplementary Materials Table S6). The density-matched neat controls recover most of the neat-to-filled softening: dilution accounts for 89–97% of the $\sigma_{y}^{0.1}$ reduction. At ϕ=0.20, the filled yield lies within the seed scatter of the density-matched neat control. At ϕ=0.30, the filled yield remains lower by 0.19–$0.22\;\varepsilon \sigma^{-3}$, indicating a secondary high-loading network contribution. We therefore attribute the de-reinforcement predominantly to the loading-induced density reduction, with the remaining high-loading contribution consistent with frozen point-LJ filler beads removing load-bearing polymer and disrupting the local chain network without adding a load-bearing filler phase.

This inverted dependence is why standard reinforcement scaling laws (Heinrich–Klüppel, power-law, percolation, Mooney) cannot describe the modulus data: each presupposes a modulus that grows with filler content, whereas the matrix softens. We therefore report the empirical de-reinforcement directly rather than forcing a reinforcement law.

The filler’s contribution is nonetheless not zero, and it is mode-selective. Whereas shear yield keeps falling monotonically through ϕ=0.30, the uniaxial yield stress recovers at ϕ=0.30 (Table 3). To test whether this recovery is a genuine filler effect or a residual of the changing matrix density, we repeated the density-matched neat-control protocol under the confined-uniaxial deformation (Table 5; per-seed values in Supplementary Materials Table S7). At ϕ=0.20, the filled uniaxial yield lies within the seed scatter of the density-matched neat control (filler contribution between −25% and +11% of the filled yield, not statistically significant at any rate), so the uniaxial response there, like the shear response, is set by matrix density. At ϕ=0.30, by contrast, the filled uniaxial yield rises well above the density-matched neat control run at the same polymer density, isolating a filler contribution of 35–42% of the filled uniaxial yield (0.34–$0.91\;\varepsilon \sigma^{-3}$) across the three rates. The contribution is positive at all three rates with a bootstrap 95% CI on the difference that excludes zero at each (CI [0.07,0.53], [0.21,0.80], and [0.65,1.18] at $\dot{\varepsilon }=10^{-4}$, $10^{-3}$, and $10^{-2}$), and it is statistically resolved at the highest rate $\dot{\varepsilon }=10^{-2}$ (filled 2.18±0.32 versus neat-density 1.27±0.12; Welch t=5.7, p=0.002), with the two slower rates corroborating the direction (Welch p≈0.09 and 0.14). The same-density comparison is diagnostic because the filled ϕ=0.30 uniaxial yield recovers above its ϕ=0.20 value at every rate (+75 to +88%), even though the matrix is more dilute and the shear yield keeps falling—so the recovery cannot be a density effect and instead isolates a load-bearing filler contribution that appears only at the highest loading and only in the deviatoric (uniaxial) mode. At ϕ=0.20, by contrast, the filler contribution is not statistically significant at any rate. The contribution is quantitatively mode-selective: at ϕ=0.30, the filler accounts for only ∼10% of the shear yield (the 89–91% dilution above) but 35–42% of the uniaxial yield. This recovery, together with the rise in $r_{y}$ (still below the von-Mises reference $\sqrt{3}$) and the shear rate-crossover, marks a qualitative change at ϕ=0.30, synthesized by the regime map and state-space trajectory in Figure 7b,c.

At shorter chain lengths N∈{25,50}, the single-seed Green–Kubo $G_{N}$ is dominated by extraction noise (adjacent-ϕ scatter of 40–90%) and no $G_{N}\left(\phi \right)$ trend is resolvable; these single-seed Direction-A data are tabulated in the Supplementary Materials (Table S3).

### 3.5. Reduced Yield Strain $\sigma_{y}/\left(G_{N}\gamma_{y}\right)$ Is Not ϕ-Independent

**Figure 8:**
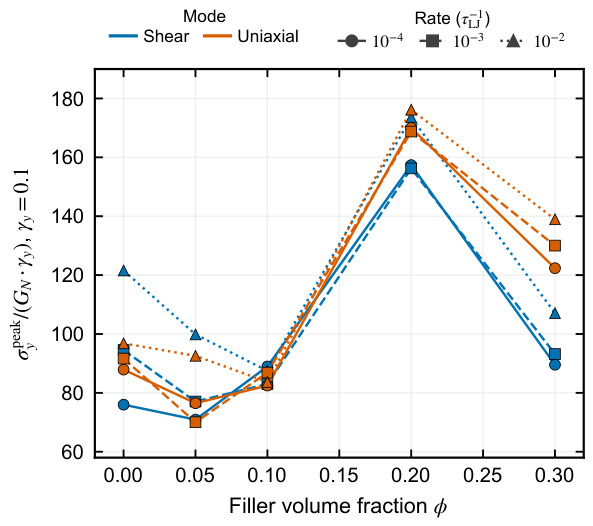
Reduced yield strain $\sigma_{y}^{\mathrm{peak}}/\left(G_{N}\gamma_{y}\right)$ versus ϕ at fixed (mode, $\dot{\varepsilon }$), with $\gamma_{y}=0.1$. The hypothesis of ϕ-independent reduced yield strain (horizontal line) is not supported: the coefficient of variation across ϕ remains large under every normalization, with the fixed-strain $\gamma_{y}=0.1$ conditions in the 0.28–0.36 range (Table 6). Linear modulus and a fixed yield-strain ratio thus do not jointly determine the nonlinear response; the strain-rate sensitivity m(ϕ) analyzed in Figure 5 acts as an independent observable.

If linear modulus $G_{N}$ and a characteristic strain $\gamma_{y}$ together fully determine the nonlinear yield response, then the dimensionless ratio $\sigma_{y}^{\mathrm{peak}}/\left(G_{N}\;\cdot \;\gamma_{y}\right)$ should be ϕ-independent at fixed (mode, $\dot{\varepsilon }$). We tested three choices of $\gamma_{y}$ (the strain at peak yield, the fixed value 0.1, and the simple ratio $\sigma_{y}/G_{N}$) across the ϕ-sweep at $T^{*}=1.0$, N=100. With every standard-rate (mode, $\dot{\varepsilon }$) condition sampled at all five ϕ with five seeds, the reduced yield strain is far from ϕ-independent under every tested normalization. For the more stable fixed-strain choice $\gamma_{y}=0.1$, the coefficient of variation across ϕ falls in the range 0.28–0.36 (Table 6 and Figure 8), implying ∼30% variation across the filler range. We therefore reject the hypothesis that linear modulus alone, combined with a fixed-yield-strain ratio, captures the nonlinear response: rate-dependent activation (cf. the m(ϕ) analysis above) operates as an independent observable orthogonal to $G_{N}$.

### 3.6. Supplementary Observations

#### 3.6.1. Temperature Dependence at ϕ=0.20

Direction-A simulations at ϕ=0.20 yield $G_{N}=0.087$ ($T^{*}=0.8$) and $G_{N}=0.034$ ($T^{*}=1.2$), a ∼2.5× drop across $\Delta T^{*}=0.4$. Because the available temperature sweep contains only the two converged endpoints, no Williams–Landel–Ferry [27] or Arrhenius fit is attempted; we retain these data as a bounded supplementary trend.

#### 3.6.2. Stretched-Exponential KWW Fits

Stretched-exponential fits (Equation (4)) to the Direction-A $\hat{G}\left(t\right)$ curves succeed ($R^{2}>0.7$) for only a subset of systems, which cluster at $\beta_{\mathrm{KWW}}\in \left[0.07,0.21\right]$, indicating broadly stretched (sub-Tg-like) relaxation. A single KWW form does not generally describe $\hat{G}\left(t\right)$ across our $\left(N,\phi ,T^{*}\right)$ matrix, so we report these successful fits in the Supplementary Materials as a supporting observation, not as a primary mechanism.

#### 3.6.3. Anomalous Shear Stress Event at ϕ=0.20, γ=−0.12

In the bidirectional shear protocol γ∈[−0.5,+0.5], a sharp ∼7σ-wide stress excursion at γ≈−0.12 is observed at ϕ=0.20, $\dot{\varepsilon }=10^{-3}$. The excursion peaks at $|\sigma_{xy}|\approx 67$ in LJ units, ∼27× the typical baseline of $|\sigma_{xy}|\approx 2.5$ at ϕ=0 under identical $\dot{\varepsilon }$. Critically, the peak location and magnitude reproduce across independent seeds (*s* = 17239, *s* = 28471), ruling out a numerical or seed-specific artifact. Outside this narrow strain window, the curves are featureless and consistent with normal inter-chain sliding. We interpret the event as a localized stress release during reverse-strain rearrangement; the monotonic forward-loading window γ∈[0,0.5] that we use for yield extraction is well outside the event and yields the normal max|σ|≈1.6 at this $\left(\phi ,\dot{\varepsilon }\right)$. We provide a diagnostic plot in Supplementary Materials Figure S1 and discuss the possible physical origin in Section 4.

## 4. Discussion

### 4.1. A Mode Asymmetry That Filler Progressively Suppresses

The central finding of this work is that uniaxial yield is more rate-sensitive than shear yield at every filler loading, most strongly in the neat-to-lightly filled melt. With the full three-rate, five-seed sampling all ten strain-rate sensitivities are resolved (uniaxial m∈[0.17,0.30], shear m∈[0.14,0.16], every CI excluding zero), and the mode gap $\Delta m=m_{\mathrm{uniax}}-m_{\mathrm{shear}}$ is positive at every loading. Its magnitude, however, is strongly loading-dependent: Δm is largest and statistically robust for ϕ≤0.10 (p(Δm>0)≥0.98) and is attenuated to within bootstrap scatter at ϕ≥0.20 (Δm≈0.02–0.04, p≈0.64–0.71). The mode asymmetry is therefore not an interphase-independent property of the two deformation geometries but a low-loading phenomenon that added filler progressively suppresses—the opposite of the naive expectation that a rigid filler network would amplify the distinction between the two modes.

We attribute the asymmetry to the differential coupling of the two deformation modes to the available molecular relaxation pathways. Under uniaxial tension, deformation is accommodated chiefly by chain stretching along the loading direction, which proceeds through a high-barrier, strongly rate-activated Eyring process [4,5,6]. Under simple shear, the same chain-stretching channel is available but is supplemented by lower-barrier inter-chain sliding and local segmental rearrangement; because these auxiliary channels carry a weaker rate dependence, the net shear rate sensitivity is lower. This picture predicts $m_{\mathrm{uniax}}>m_{\mathrm{shear}}$ at low filler loading, as observed. As filler loading increases, we hypothesize that the increasingly bridged filler network constrains the chain-stretching channel that gives uniaxial deformation its rate-sensitivity excess, which would lower $m_{\mathrm{uniax}}$ toward $m_{\mathrm{shear}}$; the mode gap does close to within statistical resolution at ϕ≥0.20, consistent with—though not proof of—this picture.

The mode gap Δm and the two-dimensional yield ratio $r_{y}$ are two projections of one loading-dependent shift in which molecular pathway accommodates yield. At low loading, low-barrier, pressure-sensitive inter-chain sliding dominates, giving $r_{y}$ near unity (well below the deviatoric-flow value $\sqrt{3}$) together with a large Δm, because uniaxial tension is forced onto the high-barrier chain-stretching channel that shear partly bypasses. Over the loading range where filler bridging saturates, Δm narrows and $r_{y}$ rises while staying below the reference $\sqrt{3}$ (the two-dimensional yield surface below); we read these as two projections of one loading-dependent shift in the operative yield pathway, a reading the co-varying descriptors support but do not, by themselves, establish.

The absolute yield stresses decrease with filler loading up to ϕ=0.20 in both modes—the shear yield $\sigma_{y}^{0.1}$ drops from ∼2.1 at ϕ=0 to ∼0.8 at ϕ=0.20 (at $\dot{\varepsilon }=10^{-3}$; see the de-reinforcement analysis above)—confirming that filler weakens yielding. That the mode gap closes precisely over the loading range where the filler-bridged network saturates is consistent with the network, rather than deformation geometry alone, shaping the high-loading rate response, although the descriptors that follow establish co-variation with loading rather than a causal link: counting, for each chain, the number of distinct filler particles it contacts (cutoff 1.5σ), the fraction of chains bridging two or more fillers rises from 61% at ϕ=0.05 to 100% at ϕ≥0.20, and the interphase fraction (polymer beads in direct filler contact) grows from 32% at ϕ=0.20 to 43% at ϕ=0.30—precisely the loading range over which the uniaxial rate sensitivity falls toward the shear value. These static descriptors co-vary with loading and with the gap closure; they locate the transition but do not by themselves probe chain stretching, inter-chain sliding, or the activated rearrangements that set the rate sensitivity, so the bridging-constraint account remains a hypothesis to be tested by direct conformational or dynamical measurements.

### 4.2. A Shear-Yield Rate Crossover at the Highest Loading

**Figure 9:**
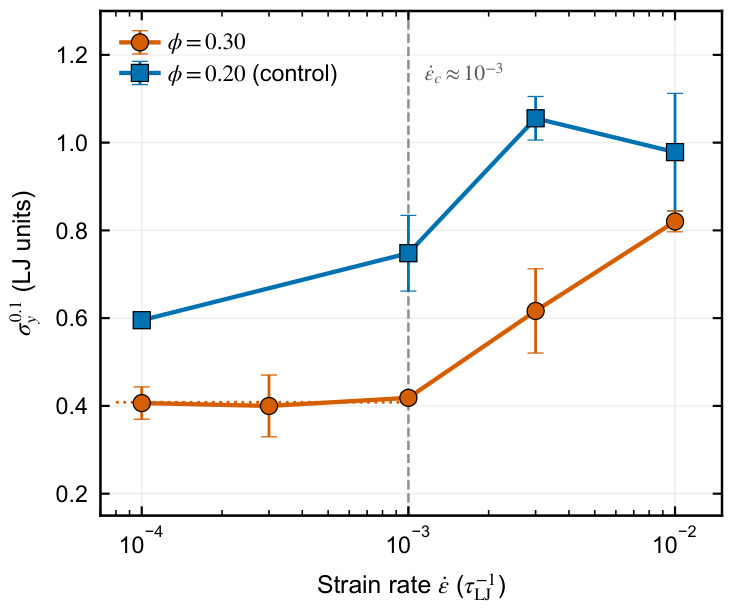
Shear small-strain yield stress $\sigma_{y}^{0.1}$ versus strain rate for ϕ=0.30 (orange, five rates) and a ϕ=0.20 control (blue, four rates), seed-averaged with error bars showing the seed-to-seed standard deviation. At ϕ=0.30, the yield is rate-independent below $\dot{\varepsilon }\approx 10^{-3}$ (dotted line at $\sigma_{y}\approx 0.41$) and rate-strengthening above it; the ϕ=0.20 control shows no comparable low-rate plateau. The intermediate rates ($3\times 10^{-4}$, $3\times 10^{-3}$) were added specifically to resolve the curve shape.

While filler suppresses the mode asymmetry rather than amplifying it, the shape of the shear $\sigma_{y}\left(\dot{\varepsilon }\right)$ response does change qualitatively at the highest loading. Resolving ϕ=0.30 with five strain rates (adding $\dot{\varepsilon }=3\times 10^{-4}$ and $3\times 10^{-3}$ to the standard three) reveals a crossover (Figure 9): the small-strain shear yield is rate-*independent* below $\dot{\varepsilon }\approx 10^{-3}$ (a plateau at $\sigma_{y}\approx 0.41$, spanning $\dot{\varepsilon }=10^{-4}$ to $10^{-3}$, local slope ≤0.04) and rate-*strengthening* above it (local slope ≈0.24–0.35). The single-Eyring *m* reported above averages over both regimes; the response is better described as bilinear. A ϕ=0.20 control does not show this low-rate plateau (its yield rises from the slowest rate through the intermediate control point), indicating that the crossover is specific to the highest, fully bridged loading in the present matrix. One possible reading is a competition between the imposed strain rate and a characteristic relaxation rate of the filler-bridged contact network (∼$10^{-3}\;\tau_{\mathrm{LJ}}^{-1}$): below such a rate the network would relax during deformation, giving quasi-static, rate-independent yield, and above it the response would become rate-strengthening. We emphasize that the seed-to-seed scatter in $\sigma_{y}$ is substantial at the intermediate rates (standard deviations up to 0.10 at $\dot{\varepsilon }=3\times 10^{-3}$), so the crossover rate is identified only to within a factor of a few.

More importantly, this network reading cannot be separated from a host-matrix origin on the present data. To probe the relevant timescale, we computed two equilibrium relaxation observables of the host at the same state point ($T^{*}=1.0$; Supplementary Materials Table S8): the Green–Kubo shear-stress autocorrelation and the monomer mean-squared displacement. At ϕ=0.30 the shear-stress autocorrelation is still ∼23% undecayed at $2.5\times 10^{3}\;\tau_{\mathrm{LJ}}$, and the monomer MSD remains sub-cage (≤$0.30\;\sigma^{2}$, below the ∼$1\;\sigma^{2}$ structural-relaxation scale) throughout $2.5\times 10^{4}\;\tau_{\mathrm{LJ}}$; the two clocks bracket the host structural relaxation at ∼$10^{3}\;\tau_{\mathrm{LJ}}$ and beyond, i.e., of order $1/{\dot{\varepsilon }}_{c}$. The crossover timescale therefore falls within the range of the host matrix’s own slow glassy relaxation, and we cannot attribute the low-rate plateau specifically to the filler-bridged network rather than to host glassy dynamics. We accordingly report the crossover as an empirical high-loading feature and treat its filler-network interpretation as one hypothesis, not an established mechanism. Separating the two would require a direct probe such as the incoherent intermediate scattering function $F_{s}\left(q,t\right)$—which the retained (positions-only) trajectories do not support—together with a finer-grained, larger-ensemble rate sweep.

It is instructive to interpret our *m* values through the activation-barrier framework. The neat values ($m_{\mathrm{shear}}=0.162$, $m_{\mathrm{uniax}}=0.243$ at $T^{*}=1.0$) are substantially larger than the weak rate sensitivity expected for fast, fluid-like segmental flow, and are instead characteristic of the cooperative, strongly activated yield of a kinetically arrested (glassy) state. This is precisely what the Section 2 diagnostics predict: the caged segmental dynamics (mean-square displacement plateauing near $0.06\;\sigma^{2}$ for the neat melt and ≈$0.08\;\sigma^{2}$ at ϕ=0.30) place the system in the activated-deformation regime, in which yield proceeds by Eyring–Argon-type barrier crossing [4,5]—the regime for which the Ree–Eyring and cooperative formulations of amorphous-polymer yield were developed [6]. The internal consistency between the elevated rate sensitivity and the independently measured caged dynamics supports the activation-barrier interpretation; we regard it as a qualitative, mechanism-level result rather than a quantitative, chemistry-specific prediction.

### 4.3. Two-Dimensional Yield Surface: Below the Continuum Reference Values at Low Loading, with a Deviatoric Contribution at ϕ=0.30

The ratio $r_{y}=\sigma_{y}^{\mathrm{uniax}}/\sigma_{y}^{\mathrm{shear}}$ falls in [0.80,1.40] across all fifteen matched $\left(\phi ,\dot{\varepsilon }\right)$ cells, in every case below the $\sqrt{3}\approx 1.73$ that the von-Mises criterion—the lower of the two isotropic-continuum bounds, with Tresca giving $r_{y}=2$—would predict for an isotropic elastic–plastic continuum. Although von-Mises ($r_{y}=\sqrt{3}$) and Tresca ($r_{y}=2$) are the bounding analytic limits for isotropic continua and our coarse-grained glassy composites are neither strictly isotropic nor strictly continuum, the observation that $r_{y}$ clusters near unity for ϕ≤0.20 ($r_{y}\in \left[0.80,1.16\right]$)—well below both bounds—is consistent with a yield process dominated by inter-chain sliding (and pressure-sensitive) rather than by bulk $J_{2}$ deviatoric stress accumulation. At the highest loading ϕ=0.30, however, $r_{y}$ rises to [1.30,1.40] (still below the von-Mises reference value $\sqrt{3}$), consistent with the high-loading bridged filler network adding a deviatoric-stress-bearing contribution that the lower-loading melts lack—a contribution isolated by the density-matched uniaxial control (Section 3.4, Table 5), in which the filled uniaxial yield at ϕ=0.30 exceeds the neat matrix at the same density (statistically resolved at $\dot{\varepsilon }=10^{-2}$). Because the uniaxial protocol imposes confined strain rather than free stress and these coarse-grained composites are neither strictly isotropic nor continuum, we use $\sqrt{3}$ and 2 only as reference bounds; the persistent shortfall below $\sqrt{3}$ even at ϕ=0.30 means we do not claim the system attains a von-Mises criterion.

Two further refinements of this interpretation are worth noting. First, the constrained-uniaxial protocol used here (the *y* and *z* box edges are held fixed during *x*-deformation) imposes a partially confined geometry that differs from free uniaxial extension; the resulting deviatoric stress proxy $\sigma_{\mathrm{dev}}=\sigma_{xx}-\frac{1}{2}\left(\sigma_{yy}+\sigma_{zz}\right)$ captures the maximum-shear-stress component but may underestimate the peak axial stress relative to a truly free-extension geometry. Second, the rate dependence of $r_{y}$ at ϕ=0.20 ($r_{y}=1.13$ at $\dot{\varepsilon }=10^{-4}$, falling to 0.93 at $\dot{\varepsilon }=10^{-2}$) suggests that filler-modulated mode coupling becomes weaker as the deformation timescale falls below the structural (α) relaxation time $\tau_{\alpha }$ of the arrested melt. This rate sensitivity of $r_{y}$ is itself a mode-coupling diagnostic and provides a more nuanced signature than the simple $r_{y}$ point estimate. We therefore propose that experimentalists who wish to compare against our results perform the shear-tension comparison at a matched Weissenberg number, $\mathrm{Wi}=\dot{\varepsilon }\tau_{\mathrm{rep}}$, rather than at matched dimensional rate, with $\tau_{\mathrm{rep}}$ estimated from the linear stress-relaxation G(t) curves.

### 4.4. De-Reinforcement and the Single-Seed Modulus Limit

The de-reinforcement of the matrix at N=100—linear $G_{N}$ and shear yield both falling with loading—deserves explicit discussion alongside its single-seed caveat. The Direction-A $G_{N}$ data carry real noise: single-seed $G_{N}$-extraction at ∼80 chains in a ∼$5000\;\sigma^{3}$ box produces fractional scatter of order 30–50% (estimated from the multi-seed Direction-B replicates), which is why we do not anchor the de-reinforcement claim on $G_{N}$ alone. The statistical weight rests instead on the multi-seed shear yield stress, which decreases strictly monotonically at every strain rate (ρ=−1.00, p<0.001); the single-seed $G_{N}$ trend is consistent in direction and corroborates it. Standard reinforcement scaling laws (Heinrich–Klüppel, predicting $R\approx 1+2.5\phi +14.1\phi^{2}$, a ∼7× growth over ϕ=0–0.30) are inapplicable not because of noise but because they presuppose modulus growth, the opposite of the observed softening. Resolving the linear modulus scaling at the shorter chain lengths, where single-seed $G_{N}$ is noise-dominated, would require multi-seed ensemble averaging (≥5 replicates per cell) and is left to future work.

### 4.5. Scope: Bead-Spring Universality Versus Industrial Polymer Mapping

The bead-spring model used here represents a chemically generic polymer melt: each bead maps notionally to 5–10 backbone monomers of a polyolefin, and the LJ length unit is mapped to σ≈0.5nm. The system therefore captures coarse-grained universality features of polymer–NP composites—e.g., filler-network reinforcement, segmental activation, and the role of chain stretching—without resolving the specific monomer chemistry of any particular industrial polymer. The m(ϕ) values reported here ($m_{\mathrm{shear}}\in \left[0.14,0.16\right]$ and $m_{\mathrm{uniax}}\in \left[0.17,0.30\right]$) should be compared with experimental values for *coarse-grained equivalent* systems rather than with measurements of specific industrial blends. Comparisons to chemistry-specific dynamic mechanical analysis of industrial polymers should therefore be framed as scale and chemistry contrasts, not as a direct validation of the present bead-spring parameters.

Available experimental studies nevertheless support the qualitative premise that polymer yield is a rate-activated observable and that filler content can modify the apparent rate response. Classic tensile experiments on glassy polymers showed that yield stress follows an Eyring-type dependence over broad temperature and strain-rate ranges [6], and large-strain compression experiments on PMMA demonstrated that rate and temperature effects can be entangled with thermomechanical softening at moderate rates [28]. For a mode-resolved comparison, Mohanraj et al. measured polyoxymethylene in uniaxial tension and simple shear at 160 °C over $10^{-4}$–$1\;s^{-1}$ and found the von-Mises equivalent yield stress to vary linearly with logarithmic strain rate [29]. For filled systems, Jančář et al. reported strain-rate- and temperature-dependent upper, lower, and rejuvenated yield stresses in PMMA micro- and nanoparticle composites [30], while Elmahdy et al. found that silica-filled RTM6 epoxy nanocomposites remain rate-sensitive in compression across $10^{-3}$–$10^{3}\;s^{-1}$, with fitted power-law rate exponents of about 0.033–0.036 [31]. The present bead-spring *m* values (0.14–0.30) lie a factor of ∼4−9 above these experimental power-law exponents; the offset is expected rather than contradictory, since the coarse-grained melt is sampled near kinetic arrest ($T^{*}=1.0$, ρ≈1.4–$1.9\;\sigma^{-3}$) at high reduced strain rates and $\sigma_{y}^{0.1}$ is a small-strain flow stress rather than a peak engineering yield—conditions that place the response deep in the activated, strongly rate-sensitive glassy regime. The comparison is thus one of regime and trend, not of absolute magnitude. These macroscopic studies provide qualitative context for rate-sensitive polymer and polymer-composite yield, but they do not supply the matched same-material shear–uniaxial $m_{\mathrm{uniax}}/m_{\mathrm{shear}}$ comparison needed to calibrate the present bead-spring model directly. The contribution of the present simulation matrix is therefore the internally matched mode comparison, rather than a claim of experimental validation.

### 4.6. Limitations

We list five principal limitations:

*(i) Single-seed Direction-A sampling.* The linear plateau modulus $G_{N}$ is extracted from single-seed Direction-A simulations and therefore corroborates, rather than independently establishes, the de-reinforcement trend; the statistical anchor is the multi-seed Direction-B shear yield stress (ρ=−1.00, p<0.001). Multi-seed Direction-A averaging would tighten the $G_{N}\left(\phi \right)$ trend, particularly at the shorter chain lengths where single-seed extraction is noise-dominated. The matched uniaxial density-matched control that isolates the filler’s mode-selective contribution to the ϕ=0.30 uniaxial recovery has now been performed (Table 5); it shows the filler contribution directly—the filled ϕ=0.30 uniaxial yield exceeds the density-matched neat melt, statistically resolved at the higher rate and corroborated at the lower—rather than leaving it inferred, though from a single filler realization (Limitation v).

*(ii) Filler-particle interaction is point-LJ.* The simulation uses $\sigma_{2-2}=1$ and $\sigma_{1-2}=1$ for filler–filler and polymer–filler LJ interactions, even though the notional filler diameter is reported as $\sigma_{f}=3.0\;\sigma$ in our earlier geometric convention [16]. The labeled ϕ is proportional to the filler number density: the filled configurations contain 35, 74, 166, and 285 filler beads for ϕ=0.05,0.10,0.20,0.30, respectively, and the filler-count-to-box-volume ratio scales linearly with the nominal label (ratios 1:1.92:3.89:6.05 versus nominal 1:2:4:6), confirming internally consistent ϕ assignment for the filled systems. Reviewers seeking strict geometric correspondence should consult the geometric calibration in [16]. This limitation is central to interpreting the negative reinforcement result: the model does not include a finite hard nanoparticle surface or a strong polymer–filler attraction that would carry additional load. The softening should therefore be read as the response of a controlled frozen, point-LJ inclusion limit, not as a general prediction that all nanofillers soften glassy polymers. Reinforcement reported for attractive or mobile nanoparticle composites [13] reflects a different interaction and geometry class.

*(iii) Neat baseline rebuilt for this study.* The ϕ=0 (filler-free) baseline reported here was generated specifically for this work by removing all filler beads from the ϕ=0.05 configuration and re-equilibrating under NPT/NVT before applying the NEMD protocols, yielding a genuine 8000-bead, zero-filler melt (Section 2). This step was taken because the filler-free configuration inherited from [16] was found to be byte-identical to the ϕ=0.05 configuration; the rebuilt neat baseline removes this redundancy and provides a true unfilled reference. The resulting neat strain-rate sensitivities ($m_{\mathrm{shear}}=0.162$, $m_{\mathrm{uniax}}=0.243$) differ negligibly from the previous low-filler estimates, confirming that the neat baseline is robust and that the low-loading mode asymmetry is unaffected.

*(iv) Interpretive caveats: confined geometry and glass assignment.* The uniaxial protocol imposes uniaxial *strain* (transverse edges fixed), not free uniaxial stress, so $\sigma_{\mathrm{dev}}$ is an exact deviatoric invariant only for an axisymmetric transverse state, held to a few percent at most loadings but departing by up to ∼46% at ϕ=0.10; the $r_{y}$ comparison to von-Mises ($\sqrt{3}$) and Tresca (2) is thus a qualitative reference, not a strict criterion test. Similarly, the “glassy” label is operational, resting on the caged-dynamics (MSD-plateau) diagnostic at $T^{*}=1.0$ rather than an independently mapped $T_{g}\left(\rho \right)$, and the ϕ=0.30 shear-rate crossover cannot be uniquely assigned to the filler-bridged network versus the host structural relaxation on the present data (see the crossover discussion above).

*(v) Single filler realization per loading.* Each ϕ is represented by one equilibrated filler configuration, and the five NEMD seeds re-draw only the initial velocities; the bootstrap confidence intervals therefore capture thermal (seed-level) variability at fixed geometry, not the configurational (filler-packing) variance that independent quenched realizations would sample. This bears on the absolute m(ϕ) values and on the cross-ϕ loading trend more than on the central mode-asymmetry result: the mode gap Δm at each loading is a *paired* comparison in which the shear and uniaxial runs act on the identical filler realization, so configurational variability largely cancels and does not generate the low-loading Δm>0 signal. Consistent with this scoping, the weakest configuration-sensitive claim—that the mode gap collapses between low and high loading—is already reported as not statistically resolved (onset p=0.11). The ϕ=0.30 filler contribution isolated by the density-matched controls (Section 3.4) is likewise an absolute, non-paired quantity—the difference between the filled and neat-density yields at one filler realization—and so, unlike Δm, is not protected by the cross-mode cancellation above; it rests on the statistically resolved $\dot{\varepsilon }=10^{-2}$ cell and the rate-consistent recovery, and a multi-realization estimate of its configurational uncertainty, together with the m(ϕ) variance, is left to future work.

### 4.7. Physical Origin of the Negative-Strain Stress Event at γ=−0.12 (ϕ=0.20, $\dot{\gamma }=10^{-3}$)

A separate but reproducible feature of our ϕ=0.20, $\dot{\gamma }=10^{-3}$ shear data is the sharp stress excursion at γ≈−0.12 during the bidirectional loading protocol. The event reproduces across independent seeds at identical strain position with comparable magnitude ($|\sigma_{xy}|\approx 67$ in LJ units, ∼27× the baseline of ∼2.5 at the same rate and neat ϕ=0). Three candidate physical mechanisms are consistent with this observation: (i) a localized chain-filler bond rearrangement during reverse-strain unloading, in which strain-induced bond realignment from the prior γ>0 excursion is suddenly released when the local stress field changes sign; (ii) a bridged filler-cluster relaxation, in which a metastable filler-chain network formed during forward shear collapses cooperatively in a single ∼$2\;\tau_{\mathrm{LJ}}$ window; or (iii) a periodic-boundary artifact specific to the tilt-box geometry at $\phi \approx \phi_{c}$ (the bridging-saturation loading). Disambiguating these requires per-bead force decomposition during the event, which is outside the scope of the present analysis. The event lies entirely outside our γ∈[0,0.5] yield-extraction window and does not affect the m(ϕ) analysis; we report it as a separate observation of interest for future cooperative-rearrangement studies in filled polymer melts.

### 4.8. Connection to Gradient Interphase and Chain Dynamics in the Same System

The gradient interphase characterization of our earlier study [16] for the same geometric protocol found a density-gradient interphase $h_{\mathrm{bound}}$ of order 1–2σ surrounding each filler particle, with chain dynamics in this interphase region slowed by a factor of ∼2−5 relative to the bulk. The present yield analysis suggests a complementary nonlinear-regime signature of the same interphase: the shear-yield rate crossover that emerges only at ϕ=0.30 coincides with the loading at which the interphase regions surrounding adjacent filler particles overlap into a fully bridged network (interphase fraction ≈43%). One might associate the crossover rate ${\dot{\varepsilon }}_{c}\approx 10^{-3}\;\tau_{\mathrm{LJ}}^{-1}$ with a relaxation time of this bridged interphase network; as shown in Section 4.2, however, the host matrix’s own glassy relaxation lies at the same timescale, so this association is one hypothesis rather than an identification. Either way, the practical implication holds: experimentalists who wish to detect an analogous crossover should target filled-polymer systems near or above an independently characterized filler-network overlap threshold and should sweep the shear-mode rate response across a wide dynamic range rather than measuring at a single rate.

## 5. Conclusions

We have surveyed the linear and large-deformation response of a Kremer–Grest polymer–nanoparticle composite at N=100 across ϕ∈[0,0.30] and three strain rates spanning two decades, using a 150-curve non-equilibrium MD matrix that closes every cell ($\dot{\varepsilon }\in \left\{10^{-4},10^{-3},10^{-2}\right\}$) at five independent thermal-velocity seeds in both modes across all five filler concentrations. The strain-rate sensitivity $m\left(\phi \right)=\partial \ln\sigma_{y}^{0.1}/\partial \ln\dot{\varepsilon }$ provides the most statistically resolved nonlinear observable in our data: with a full three-rate, five-seed sampling, uniaxial m∈[0.17,0.30] and shear m∈[0.14,0.16] are all resolved (every CI excluding zero), and the uniaxial–shear mode gap, while positive at every loading, is strongly loading-dependent—largest and statistically robust at low loading (ϕ≤0.10) and attenuated to within bootstrap scatter at ϕ≥0.20. The mode asymmetry is thus a low-loading phenomenon that added filler progressively suppresses rather than a loading-invariant geometric effect. Separately, at the highest loading, the shear yield exhibits a rate crossover—rate-independent below $\dot{\varepsilon }\approx 10^{-3}$ and rate-strengthening above—which we tentatively associate with the relaxation rate of the bridged filler network, though a host-matrix glassy-relaxation origin cannot be excluded on the present data. The two-dimensional yield-stress ratio $\sigma_{y}^{\mathrm{uniax}}/\sigma_{y}^{\mathrm{shear}}$ rises from a near-parity [0.80,1.16] for ϕ≤0.20 (well below both the von-Mises and Tresca continuum reference values) to [1.30,1.40] at ϕ=0.30, remaining below the von-Mises reference. Consistent with these signatures, frozen filler de-reinforces the matrix at N=100—both the linear plateau modulus and the shear yield stress decrease monotonically with loading (shear $\sigma_{y}$ by ∼78%, ρ=−1.00; $G_{N}$ by ∼43%), a softening for which density-matched neat controls recover 89–97% of the reduction, consistent with dominant matrix-density dilution, while density-matched controls run in both deformation modes isolate a genuine, mode-selective filler contribution at ϕ=0.30 (35–42% of the uniaxial yield versus about 10% of the shear yield)—so standard reinforcement scaling laws, which presuppose modulus growth, do not apply. The reduced yield strain $\sigma_{y}/\left(G_{N}\gamma_{y}\right)$ varies by ∼30% across ϕ, ruling out a simple linear-modulus control of nonlinear yield. The mode-gap attenuation, the shear rate-crossover, the rise of $r_{y}$ (staying below the von-Mises reference $\sqrt{3}$), and the mode-selective filler contribution isolated by the density-matched controls all converge at ϕ=0.30, pointing to a qualitative change in load transfer once the filler network becomes fully bridged. The mode-selective m(ϕ) analysis operates as an observable orthogonal to the linear plateau modulus and provides a new mechanistic window into how filler loading modulates the activation pathway of polymer-melt yield.

## Figures and Tables

**Table 1:** Dataset overview (after 2026-05-28 supplementary runs). Numbers in parentheses are the number of independent seeds.

Block	*N*	ϕ	$\dot{\varepsilon }$ ($\tau_{\mathrm{LJ}}^{-1}$)	Systems (Seeds)
Direction-A plateau ($T^{*}=1.0$)	25, 50, 100	0.05–0.40	—	20 (1)
Direction-A *T*-sweep support	100	0.20	—	2 extra ($T^{*}=0.8,1.2$)
Direction-B shear	100	0.00–0.30	$10^{-4},10^{-3},10^{-2}$	75 (5)
Direction-B uniaxial	100	0.00–0.30	$10^{-4},10^{-3},10^{-2}$	75 (5)

**Table 2:** Strain-rate sensitivity $m=\partial \ln\sigma_{y}^{0.1}/\partial \ln\dot{\varepsilon }$ with 95% bootstrap CI from 1000 Monte Carlo iterations of $\sigma_{y}∼N\left(\mathrm{mean},\mathrm{std}\right)$, with std the five-seed standard deviation at each strain rate.

	ϕ=0.00	ϕ=0.05	ϕ=0.10	ϕ=0.20	ϕ=0.30
Shear *m*	0.162	0.146	0.149	0.146	0.147
95% CI	[0.133, 0.189]	[0.087, 0.198]	[0.081, 0.231]	[0.015, 0.229]	[0.033, 0.268]
Uniaxial *m*	0.243	0.301	0.265	0.186	0.170
95% CI	[0.163, 0.313]	[0.242, 0.358]	[0.192, 0.328]	[0.025, 0.294]	[0.095, 0.225]
Mode gap Δm	+0.081	+0.155	+0.116	+0.040	+0.023

**Table 3:** Two-dimensional yield surface: peak-yield ratio $r_{y}=\sigma_{y}^{\mathrm{uniax}}/\sigma_{y}^{\mathrm{shear}}$. Shear $\sigma_{y}$ is $\max|\sigma_{xy}|$ in γ∈[0,0.5]; uniaxial $\sigma_{y}$ is $\max|\sigma_{\mathrm{dev}}|$ in the same strain window.

ϕ	$\dot{\varepsilon }$ ($\tau_{\mathrm{LJ}}^{-1}$)	$\sigma_{y}^{\mathrm{shear}}$	$\sigma_{y}^{\mathrm{uniax}}$	$r_{y}$
0.00	$10^{-4}$	2.15	2.49	1.16
0.00	$10^{-3}$	2.67	2.59	0.97
0.00	$10^{-2}$	3.44	2.74	0.80
0.05	$10^{-4}$	2.01	2.16	1.08
0.05	$10^{-3}$	2.18	1.98	0.91
0.05	$10^{-2}$	2.83	2.62	0.93
0.10	$10^{-4}$	2.09	1.94	0.93
0.10	$10^{-3}$	1.95	2.04	1.05
0.10	$10^{-2}$	2.05	1.97	0.96
0.20	$10^{-4}$	1.36	1.46	1.08
0.20	$10^{-3}$	1.35	1.45	1.08
0.20	$10^{-2}$	1.52	1.52	1.00
0.30	$10^{-4}$	1.43	1.96	1.37
0.30	$10^{-3}$	1.49	2.08	1.40
0.30	$10^{-2}$	1.71	2.22	1.30

**Table 4:** Density-matched neat controls separating dilution from a filler-network effect. Small-strain shear yield $\sigma_{y}^{0.1}$ ($\varepsilon \sigma^{-3}$) for the full-density neat melt, a neat melt compressed to the polymer density of the filled box, and the filled system, at the two highest loadings. “Dilution” is the fraction of the neat-to-filled softening recovered by the density-matched neat control. Each density-control cell uses three independent seeds (mean ± s.d.; Supplementary Materials Table S6).

ϕ	$\dot{\varepsilon }$ ($\tau_{\mathrm{LJ}}^{-1}$)	Neat ($\rho_{\mathrm{full}}$)	Neat ($\rho_{\mathrm{matched}}$)	Filled	Dilution
0.20	$10^{-3}$	2.10	0.83±0.03	0.78±0.08	96%
0.20	$10^{-2}$	3.09	1.27±0.19	1.22±0.28	97%
0.30	$10^{-3}$	2.10	0.59±0.09	0.40±0.05	89%
0.30	$10^{-2}$	3.09	0.91±0.10	0.69±0.12	91%

**Table 5:** Density-matched neat controls under confined-uniaxial extension, isolating the filler’s deviatoric contribution, at the two highest loadings and all three strain rates. Small-strain uniaxial yield $\sigma_{y}^{0.1}$ ($\varepsilon \sigma^{-3}$) for the full-density neat melt, a neat melt compressed to the polymer density of the filled box, and the filled system (mean ± s.d.; three seeds for the neat controls, five for the filled; Supplementary Materials Table S7). The filler contribution is (filled − density-matched neat); at ϕ=0.30, it is positive with bootstrap 95% CI excluding zero at every rate, and, at ϕ=0.20, it is not significant at any rate.

ϕ	$\dot{\varepsilon }$ ($\tau_{\mathrm{LJ}}^{-1}$)	Neat ($\rho_{\mathrm{full}}$)	Neat ($\rho_{\mathrm{matched}}$)	Filled	Filler Contribution
0.20	$10^{-4}$	0.84	0.66±0.17	0.53±0.07	−0.13 (−25%)
0.20	$10^{-3}$	1.26	0.65±0.11	0.73±0.13	+0.08 (+11%)
0.20	$10^{-2}$	2.56	1.33±0.28	1.25±0.33	−0.08 (−6%)
0.30	$10^{-4}$	0.84	0.65±0.25	1.00±0.03	+0.34 (+35%)
0.30	$10^{-3}$	1.26	0.77±0.32	1.32±0.10	+0.55 (+42%)
0.30	$10^{-2}$	2.56	1.27±0.12	2.18±0.32	+0.91 (+42%)

**Table 6:** Coefficient of variation of $\sigma_{y}^{\mathrm{peak}}/\left(G_{N}\;\cdot \;\gamma_{y}\right)$ across ϕ at fixed (mode, $\dot{\varepsilon }$), using $\gamma_{y}=0.1$ (the more stable choice). Smaller CV would indicate ϕ-independence.

Mode	$\dot{\varepsilon }$ ($\tau_{\mathrm{LJ}}^{-1}$)	CV Across ϕ
Shear	$10^{-4}$	0.362
Shear	$10^{-3}$	0.316
Shear	$10^{-2}$	0.284
Uniaxial	$10^{-4}$	0.362
Uniaxial	$10^{-3}$	0.363
Uniaxial	$10^{-2}$	0.332

## Data Availability

All LAMMPS input scripts, the five distinct equilibrated starting configurations (with MD5 checksums), aggregated stress–strain data, processed figure data tables, and the complete Python analysis scripts (aggregation, yield extraction, Eyring and paired-bootstrap fitting, figure generation) will be deposited in a public Zenodo repository upon acceptance and are available to editors and reviewers during peer review.

## Supplementary Materials

The following supporting information can be downloaded at: https://www.mdpi.com/article/10.3390/polym18151823/s1: Figure S1: Reverse-strain shear-stress-spike diagnostic at ϕ=0.20, $\dot{\gamma }=10^{-3}$; Table S1: Per-cell yield-stress extraction summary ($\sigma_{y}^{\mathrm{peak}}$ and $\sigma_{y}^{0.1}$) for all standard-rate NEMD cells; Table S2: MD5 checksums and structural fingerprints ($N_{\mathrm{filler}}$, ρ, $R_{g}$) of the five distinct starting configurations; Table S3: Single-seed Direction-A reinforcement factor $R_{\mathrm{reinforce}}\left(N,\phi \right)$; Table S4: Peak-yield Eyring fits; Table S5: Successful KWW fits; Table S6: Density-matched neat control yields (shear); Table S7: Density-matched neat control yields under confined-uniaxial extension; Table S8: Host-matrix equilibrium relaxation observables. The complete simulation archive—all 150 standard stress–strain curves, the six intermediate-rate crossover runs, the density-matched neat controls, the Direction-A G(t) relaxation curves and KWW fits, the LAMMPS input decks, and the Python analysis scripts—will be deposited in a public Zenodo repository upon acceptance and is available to editors and reviewers during peer review.

## Abbreviations

MD	Molecular Dynamics
NEMD	Non-Equilibrium Molecular Dynamics
CG	Coarse-Grained
K–G	Kremer–Grest
SLLOD	Gaussian Isokinetic SLLOD Algorithm
LJ	Lennard–Jones
KWW	Kohlrausch–Williams–Watts
CI	Confidence Interval

## References

[1] Heinrich G., Klüppel M., Vilgis T.A. (2002). Reinforcement of Elastomers. Curr. Opin. Solid State Mater. Sci..

[2] Kalathi J.T., Grest G.S., Kumar S.K. (2012). Universal Viscosity Behavior of Polymer Nanocomposites. Phys. Rev. Lett..

[3] Vilgis T.A., Heinrich G., Klüppel M. (2009). Reinforcement of Polymer Nano-Composites: Theory, Experiments and Applications.

[4] Eyring H. (1936). Viscosity, Plasticity, and Diffusion as Examples of Absolute Reaction Rates. J. Chem. Phys..

[5] Argon A.S. (1973). A Theory for the Low-Temperature Plastic Deformation of Glassy Polymers. Phil. Mag..

[6] Bauwens-Crowet C., Bauwens J.C., Homès G. (1969). Tensile Yield-Stress Behavior of Glassy Polymers. J. Polym. Sci. Part A-2 Polym. Phys..

[7] Karatrantos A., Clarke N., Kröger M. (2016). Modeling of Polymer Structure and Conformations in Polymer Nanocomposites from Atomistic to Mesoscale: A Review. Polym. Rev..

[8] Likhtman A.E., McLeish T.C.B. (2002). Quantitative Theory for Linear Dynamics of Linear Entangled Polymers. Macromolecules.

[9] Likhtman A.E., Sukumaran S.K., Ramirez J. (2007). Linear Viscoelasticity from Molecular Dynamics Simulation of Entangled Polymers. Macromolecules.

[10] Hoy R.S., Robbins M.O. (2008). Strain Hardening in Polymer Glasses: Limitations of Network Models. Phys. Rev. Lett..

[11] Todd B.D., Daivis P.J. (2007). Homogeneous non-equilibrium molecular dynamics simulations of viscous flow: Techniques and applications. Mol. Simul..

[12] Xu T., Nie C., Peng F., Sheng J., Li L. (2022). Bond Orientation-Assisted Enthalpic Stress in Polymer Glasses: A Simulation Study on Elastic Yielding. Macromolecules.

[13] Shi R., Yu L., Zhang N., Yang Y., Lu Z.Y., Qian H.J. (2023). Molecular Origin of the Reinforcement Effect and Its Strain-Rate Dependence in Polymer Nanocomposite Glass. ACS Macro Lett..

[14] Kalghatgi S.R., Kumar S.K., Mani E. (2024). Molecular Dynamics Study of the Role of Nanoparticle Assemblies on Polymer Nanocomposite Rheology. Macromolecules.

[15] Wang Y., Liu H., Li P., Wang L. (2022). The Effect of Cross-Linking Type on EPDM Elastomer Dynamics and Mechanical Properties: A Molecular Dynamics Simulation Study. Polymers.

[16] Sun Y., Wang H., Hou P., Bai W., Chu D., Deng W. (2026). Bimodal Interphase Architecture in Filled Elastomers: Molecular Dynamics Evidence and Experimental Signatures. Molecules.

[17] Sun Y., Deng W., Wang H., Jian R., Bai W., Chu D., Hou P., He Y. (2026). Machine-Learning-Assisted Viscoelastic Characterization of PC/ABS Blends via Multi-Frequency Dynamic Mechanical Analysis. Polymers.

[18] Plimpton S. (1995). Fast Parallel Algorithms for Short-Range Molecular Dynamics. J. Comput. Phys..

[19] Thompson A.P., Aktulga H.M., Berger R., Bolintineanu D.S., Brown W.M., Crozier P.S., in ’t Veld P.J., Kohlmeyer A., Moore S.G., Nguyen T.D. (2022). LAMMPS—A Flexible Simulation Tool for Particle-Based Materials Modeling at the Atomic, Meso, and Continuum Scales. Comput. Phys. Commun..

[20] Kremer K., Grest G.S. (1990). Dynamics of Entangled Linear Polymer Melts: A Molecular-Dynamics Simulation. J. Chem. Phys..

[21] Grest G.S., Kremer K. (1986). Molecular dynamics simulation for polymers in the presence of a heat bath. Phys. Rev. A.

[22] Everaers R., Sukumaran S.K., Grest G.S., Svaneborg C., Sivasubramanian A., Kremer K. (2004). Rheology and Microscopic Topology of Entangled Polymeric Liquids. Science.

[23] Evans D.J., Morriss G.P. (1984). Non-Newtonian Molecular Dynamics. Comput. Phys. Rep..

[24] Lees A.W., Edwards S.F. (1972). The Computer Study of Transport Processes Under Extreme Conditions. J. Phys. C Solid State Phys..

[25] Efron B., Tibshirani R.J. (1993). An Introduction to the Bootstrap.

[26] Williams G., Watts D.C. (1970). Non-Symmetrical Dielectric Relaxation Behaviour Arising from a Simple Empirical Decay Function. Trans. Faraday Soc..

[27] Williams M.L., Landel R.F., Ferry J.D. (1955). The Temperature Dependence of Relaxation Mechanisms in Amorphous Polymers and Other Glass-forming Liquids. J. Am. Chem. Soc..

[28] Arruda E.M., Boyce M.C., Jayachandran R. (1995). Effects of Strain Rate, Temperature and Thermomechanical Coupling on the Finite Strain Deformation of Glassy Polymers. Mech. Mater..

[29] Mohanraj J., Barton D.C., Ward I.M., Dahoun A., Hiver J.M., G’Sell C. (2006). Plastic Deformation and Damage of Polyoxymethylene in the Large Strain Range at Elevated Temperatures. Polymer.

[30] Jančář J., Hoy R.S., Jančářová E., Žídek J. (2015). Effect of Temperature, Strain Rate and Particle Size on the Yield Stresses and Post-Yield Strain Softening of PMMA and Its Composites. Polymer.

[31] Elmahdy A., Zotti A., Zuppolini S., Zarrelli M., Borriello A., Verleysen P. (2021). Effect of Strain Rate and Silica Filler Content on the Compressive Behavior of RTM6 Epoxy-Based Nanocomposites. Polymers.

