# Supplementary material for: Mode-Dependent Strain-Rate Sensitivity of Filled Polymer Yield Under Shear and Uniaxial Deformation
Source: Polymers (Basel). 2026 Jul 25;18(15):1823. doi: 10.3390/polym18151823 (PMC13468397; doi:10.3390/polym18151823)
Supplement: Supplementary file 1 [file polymers-18-01823-s001.zip › polymers_SI.pdf]

# Supporting Information

## Mode-Dependent Strain-Rate Sensitivity of Filled Polymer Yield under Shear and Uniaxial Deformation

Yancai Sun, Feng Shi, Jian Xu, Peiwu Hou,
Wenjuan Bai, Dianming Chu, Wenzhong Deng

## Figure S1. Reverse-strain stress-spike diagnostic

At $\phi = 0.20$, $\dot{\gamma} = 10^{-3}$ the raw shear-stress trace contains a large, sharp transient at engineering strain $\gamma \approx -0.12$, reaching $|\sigma_{xy}| \approx 67\,\varepsilon\sigma^{-3}$ (Figure S1). The event is confined to the reverse-strain region ($\gamma < 0$) and reproduces at an identical strain position across independent seeds (17239 and 28471), identifying it as a deterministic, localized stress-release rearrangement rather than stochastic noise. Because all yield quantities in the main text are extracted from the monotonic forward-loading window $\gamma \in [0, 0.5]$ (vertical dashed line), the transient does not enter the reported yield statistics; the forward yield at this cell is the ordinary $\max |\sigma_{xy}| \approx 1.6\,\varepsilon\sigma^{-3}$.

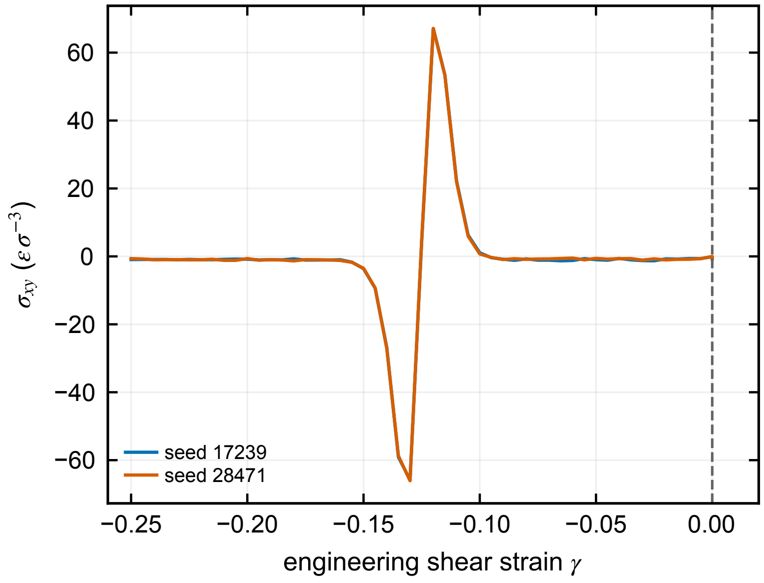


Figure S1: Shear-stress trace $\sigma_{xy}(\gamma)$ in the reverse-strain region for the two seeds at $\phi = 0.20$, $\dot{\gamma} = 10^{-3}$. The $\gamma \geq 0$ forward window used for all yield extraction lies to the right of the dashed line.

## Table S1. Per-cell yield-stress extraction summary

Seed-averaged peak yield $\sigma_y^{\mathrm{peak}}$ (maximum $|\sigma|$ in the forward-loading window $\gamma \in [0, 0.5]$) and small-strain yield $\sigma_y^{0.1}$ (mean $|\sigma|$ over $\gamma \in [0.08, 0.12]$) for all standard-rate NEMD cells, each averaged over five independent seeds.

| Mode | $\phi$ | $\dot{\varepsilon}$ ($\tau_{\mathrm{LJ}}^{-1}$) | $\sigma_y^{\mathrm{peak}}$ | $\sigma_y^{0.1}$ | Seeds |
|---|---|---|---|---|---|
| shear | 0.00 | $10^{-4}$ | 2.150 | 1.462 | 5 |
| shear | 0.00 | $10^{-3}$ | 2.675 | 2.097 | 5 |
| shear | 0.00 | $10^{-2}$ | 3.439 | 3.086 | 5 |
| shear | 0.05 | $10^{-4}$ | 2.006 | 1.195 | 5 |
| shear | 0.05 | $10^{-3}$ | 2.180 | 1.650 | 5 |
| shear | 0.05 | $10^{-2}$ | 2.825 | 2.341 | 5 |
| shear | 0.10 | $10^{-4}$ | 2.090 | 0.809 | 5 |
| shear | 0.10 | $10^{-3}$ | 1.950 | 1.072 | 5 |
| shear | 0.10 | $10^{-2}$ | 2.055 | 1.608 | 5 |
| shear | 0.20 | $10^{-4}$ | 1.355 | 0.620 | 5 |
| shear | 0.20 | $10^{-3}$ | 1.345 | 0.775 | 5 |
| shear | 0.20 | $10^{-2}$ | 1.520 | 1.215 | 5 |
| shear | 0.30 | $10^{-4}$ | 1.433 | 0.352 | 5 |
| shear | 0.30 | $10^{-3}$ | 1.491 | 0.397 | 5 |
| shear | 0.30 | $10^{-2}$ | 1.713 | 0.692 | 5 |
| uniaxial | 0.00 | $10^{-4}$ | 2.487 | 0.839 | 5 |
| uniaxial | 0.00 | $10^{-3}$ | 2.593 | 1.256 | 5 |
| uniaxial | 0.00 | $10^{-2}$ | 2.739 | 2.563 | 5 |
| uniaxial | 0.05 | $10^{-4}$ | 2.164 | 0.544 | 5 |
| uniaxial | 0.05 | $10^{-3}$ | 1.981 | 0.997 | 5 |
| uniaxial | 0.05 | $10^{-2}$ | 2.619 | 2.175 | 5 |
| uniaxial | 0.10 | $10^{-4}$ | 1.938 | 0.552 | 5 |
| uniaxial | 0.10 | $10^{-3}$ | 2.041 | 0.967 | 5 |
| uniaxial | 0.10 | $10^{-2}$ | 1.967 | 1.870 | 5 |
| uniaxial | 0.20 | $10^{-4}$ | 1.464 | 0.532 | 5 |
| uniaxial | 0.20 | $10^{-3}$ | 1.453 | 0.729 | 5 |
| uniaxial | 0.20 | $10^{-2}$ | 1.517 | 1.250 | 5 |
| uniaxial | 0.30 | $10^{-4}$ | 1.957 | 0.999 | 5 |
| uniaxial | 0.30 | $10^{-3}$ | 2.080 | 1.321 | 5 |
| uniaxial | 0.30 | $10^{-2}$ | 2.223 | 2.184 | 5 |

## Table S2. Configuration fingerprints

The five physically distinct initial bead-spring configurations, with MD5 checksums (first twelve hexadecimal digits) confirming pairwise distinctness. The filler-free configuration inherited from the parent study was byte-identical to the $\phi = 0.05$ configuration and was replaced by a purpose-built neat configuration. The filler number density scales linearly with the nominal label (ratios 1 : 1.92 : 3.89 : 6.05 versus nominal 1 : 2 : 4 : 6).

| Config | $\phi$ | Fillers | Beads | $\rho$ ($\sigma^{-3}$) | $R_g$ ($\sigma$) | MD5 (12) |
|---|---|---|---|---|---|---|
| neat | 0.00 | 0 | 8000 | 1.93 | 9.26 | `3d0c0ffde683` |
| filled | 0.05 | 35 | 8035 | 1.86 | 9.74 | `f950c1b64069` |
| filled | 0.10 | 74 | 8074 | 1.69 | 9.94 | `5d1ea8fd85f4` |
| filled | 0.20 | 166 | 8166 | 1.55 | 10.84 | `aeb67f14f0c1` |
| filled | 0.30 | 285 | 8285 | 1.42 | 12.44 | `144ad36538e3` |

## Table S3. Single-seed Direction-A reinforcement factor

The plateau-modulus reinforcement factor $R_{\text{reinforce}}(N, \phi) = G_N(\phi)/G_N(\phi = 0.05)$ from single-seed Direction-A Green–Kubo simulations at $T^* = 1.0$. At the survey chain length $N = 100$ the factor descends with loading (de-reinforcement; see main-text Figure 7); at the shorter chain lengths $N \in \{25, 50\}$ single-seed extraction is noise-dominated (adjacent-$\phi$ scatter of 40–90%) and no $\phi$-trend is resolvable.

| $N$ | $\phi = 0.05$ | $\phi = 0.10$ | $\phi = 0.15$ | $\phi = 0.20$ | $\phi = 0.25$ | $\phi = 0.30$ |
|---|---|---|---|---|---|---|
| 25 | 1.00 | 2.11 | 0.97 | 2.52 | 0.99 | 1.90 |
| 50 | 1.00 | 2.15 | 0.85 | 0.96 | 1.57 | 2.19 |
| 100 | 1.00 | 0.83 | 0.60 | 0.30 | 0.86 | 0.57 |

## Table S4. Peak-yield Eyring fits

The peak-yield observable $\sigma_y^{\text{peak}}$ was fitted with the same three-rate, five-seed bootstrap protocol used for $\sigma_y^{0.1}$ in the main text. The peak-yield slopes are generally smaller and less consistently resolved, supporting the use of $\sigma_y^{0.1}$ as the primary rate-sensitivity observable.

| Mode | $\phi$ | $m_{\text{peak}}$ (95% CI) | $R^2$ |
|---|---|---|---|
| shear | 0.00 | 0.102 [0.066, 0.134] | 0.99 |
| shear | 0.05 | 0.075 [0.037, 0.108] | 0.90 |
| shear | 0.10 | −0.004 [−0.054, 0.041] | 0.06 |
| shear | 0.20 | 0.025 [−0.035, 0.076] | 0.71 |
| shear | 0.30 | 0.039 [−0.047, 0.108] | 0.90 |
| uniaxial | 0.00 | 0.021 [−0.028, 0.065] | 0.99 |
| uniaxial | 0.05 | 0.041 [−0.007, 0.086] | 0.48 |
| uniaxial | 0.10 | 0.002 [−0.071, 0.077] | 0.07 |
| uniaxial | 0.20 | 0.007 [−0.041, 0.061] | 0.60 |
| uniaxial | 0.30 | 0.028 [−0.037, 0.080] | 1.00 |

## Table S5. Successful KWW fits

Kohlrausch–Williams–Watts (KWW) fits are listed only for cases with $R^2 > 0.7$. The fitted $\tau_{\text{KWW}}$ values sit at the lower bound of the fitting interval in these cases, so the parameters are used only as descriptive supporting data and not as a primary mechanistic observable.

| $N$ | $\phi$ | $T^*$ | $\tau_{\text{KWW}}$ | $\beta_{\text{KWW}}$ | $A_{\text{plateau}}$ | $R^2$ |
|---|---|---|---|---|---|---|
| 50 | 0.25 | 1.5 | 0.100 | 0.067 | 0.000 | 0.86 |
| 50 | 0.25 | 0.6 | 0.100 | 0.211 | 0.179 | 0.77 |
| 50 | 0.35 | 1.0 | 0.100 | 0.193 | 0.246 | 0.75 |
| 50 | 0.40 | 1.0 | 0.100 | 0.196 | 0.256 | 0.73 |
| 100 | 0.25 | 1.0 | 0.100 | 0.204 | 0.250 | 0.72 |

## Table S6. Density-matched neat control yields

Small-strain shear yield $\sigma_y^{0.1}$ ($\varepsilon\sigma^{-3}$) for neat polymer compressed to the polymer density of the $\phi = 0.20$ and $\phi = 0.30$ filled boxes, used to separate the dilution contribution from a genuine filler-network effect (main-text density-matched control table). Each reported density-control cell uses three independent seeds.

| density-matched $\phi$ | $\dot{\varepsilon}$ ($\tau_{\mathrm{LJ}}^{-1}$) | seeds | $\sigma_y^{0.1}$ |
|---|---|---|---|
| 0.20 | $10^{-3}$ | 3 | $0.83 \pm 0.03$ |
| 0.20 | $10^{-2}$ | 3 | $1.27 \pm 0.19$ |
| 0.30 | $10^{-3}$ | 3 | $0.59 \pm 0.09$ |
| 0.30 | $10^{-2}$ | 3 | $0.91 \pm 0.10$ |

## Table S7. Density-matched neat controls under uniaxial extension

Small-strain uniaxial yield $\sigma_y^{0.1}$ ($\varepsilon\sigma^{-3}$, deviatoric proxy $\sigma_{\mathrm{dev}}$) for a neat melt compressed to the polymer density of the $\phi = 0.20$ and $\phi = 0.30$ filled boxes and deformed under the same confined-uniaxial protocol as the filled runs, listed per seed, at both densities and all three strain rates. These controls, the uniaxial counterpart of the shear controls in Table S6, isolate the filler's deviatoric contribution: at $\phi = 0.20$ the density-matched neat yield matches the filled yield within seed scatter (filler contribution not significant at any rate), whereas at $\phi = 0.30$ the filled uniaxial yield rises well above the density-matched neat control at the same density, leaving a filler contribution of 35–42% of the filled uniaxial yield that is positive with a bootstrap 95% CI on the difference excluding zero at all three rates (CI $[0.07, 0.53]$, $[0.21, 0.80]$, $[0.65, 1.18]$ at $\dot{\varepsilon} = 10^{-4}$, $10^{-3}$, $10^{-2}$). It is statistically resolved at $10^{-2}$ (Welch $t = 5.7$, $p = 0.002$), with the two slower rates corroborating the direction ($p \approx 0.09$ and $0.14$). The recovery cannot be a density effect: the filled $\phi = 0.30$ uniaxial yield rises above its $\phi = 0.20$ value at every rate even though the $\phi = 0.30$ matrix is the more dilute (for which the shear yield, by contrast, keeps falling).

| density-matched $\phi$ | $\dot{\varepsilon}$ ($\tau_{\mathrm{LJ}}^{-1}$) | $s$=17239 | $s$=28471 | $s$=39827 | mean ± s.d. |
|---|---|---|---|---|---|
| 0.20 | $10^{-4}$ | 0.527 | 0.858 | 0.605 | $0.66 \pm 0.17$ |
| 0.20 | $10^{-3}$ | 0.740 | 0.524 | 0.671 | $0.65 \pm 0.11$ |
| 0.20 | $10^{-2}$ | 1.113 | 1.645 | 1.222 | $1.33 \pm 0.28$ |
| 0.30 | $10^{-4}$ | 0.939 | 0.556 | 0.468 | $0.65 \pm 0.25$ |
| 0.30 | $10^{-3}$ | 1.135 | 0.555 | 0.615 | $0.77 \pm 0.32$ |
| 0.30 | $10^{-2}$ | 1.392 | 1.148 | 1.283 | $1.27 \pm 0.12$ |

## Table S8. Host-matrix equilibrium relaxation observables

Independent relaxation clocks for the host matrix at $T^* = 1.0$, computed from the equilibrium Green–Kubo shear-stress autocorrelation function $\hat{G}(t)$ and the monomer mean-squared displacement (MSD) over the Direction-A $\phi$-sweep. "SACF residual" is the fraction of $\hat{G}(0)$ still undecayed at the $2.5 \times 10^3\,\tau_{\mathrm{LJ}}$ window edge; $\tau_G$ is the corresponding truncated integral relaxation time (a lower bound); $\mathrm{MSD}_{\mathrm{max}}$ is the monomer MSD reached at $2.5 \times 10^4\,\tau_{\mathrm{LJ}}$ (all values below the $\sim 1\,\sigma^2$ structural-relaxation scale, i.e. the host never escapes its cage within the window). At the crossover loading $\phi = 0.30$ the two observables bracket the host structural relaxation at $\sim 10^3\,\tau_{\mathrm{LJ}} \approx 1/\dot{\varepsilon}_c$ and beyond, so the $\phi = 0.30$ shear-yield crossover falls within the range of the host's own glassy relaxation and cannot be assigned uniquely to the filler network (main text, Section 4.2).

| $\phi$ | SACF residual at $2.5{\times}10^3\,\tau_{\rm LJ}$ | $\tau_G$ ($\geq$, $\tau_{\rm LJ}$) | $\mathrm{MSD}_{\max}$ ($\sigma^2$) | reaches $1\,\sigma^2$ |
|---|---|---|---|---|
| 0.05 | 16% | 433 | 0.17 | no |
| 0.10 | 6% | 172 | 0.17 | no |
| 0.15 | 10% | 287 | 0.24 | no |
| 0.25 | 22% | 562 | 0.29 | no |
| 0.30 | 23% | 590 | 0.30 | no |

## Additional supporting data

The complete simulation archive will be deposited in a public Zenodo repository upon acceptance and is available to editors and reviewers during peer review. The archive contains all 150 standard NEMD stress–strain curves, the six intermediate-rate shear runs resolving the rate crossover, the Direction-A Green–Kubo $G(t)$ relaxation curves and KWW fits, the LAMMPS input decks, the equilibrated starting configurations, and the Python analysis scripts for aggregation, yield extraction, Eyring fitting, paired-bootstrap analysis, and figure generation.
